# Exposed yet unmapped: evidence of differential flood exposure in deprived urban areas using citizen science

**DOI:** 10.1038/s42949-026-00428-8

**Published:** 2026-06-22

**Authors:** Lorraine Trento Oliveira, Florencio V. Campomanes, Anne M. Dijkstra, Mariana Belgiu, Prosper Adiku, Nicera Wanjiru, Lizian Onyango, Renaldo Flor, Deyril Ibraimo, Bastian van den Bout, Jaap A. Zevenbergen, Monika Kuffer

**Affiliations:** 1 University of Twente, Enschede, The Netherlands; 2 University of Ghana, Accra, Ghana; 3 SDI Kenya, Nairobi, Kenya; 4 Data4Moz, Maputo, Mozambique

**Keywords:** Hydrology, Developing world, Climate-change impacts, Geography

## Abstract

Rapid urbanization in Sub-Saharan Africa (SSA) has intensified flood risks, disproportionately affecting deprived urban areas (DUAs). Yet, these areas remain systematically unmapped in existing assessments, particularly in data-scarce environments. This study is among the first to center DUAs within a comparative flood exposure assessment across SSA cities, while integrating a lightweight low-cost flood model, global remote sensing datasets and citizen science methods. We find that DUAs are up to 200% more exposed to flooding, with frequent shallow floods (up to 10 cm) causing significant, yet often overlooked, impacts. Of these, over 50% concern property damage, disease outbreaks and infrastructure failures. Surprisingly, secondary cities sometimes surpass primaries in absolute exposure. We also highlight the spatial biases from built-up surface datasets, in turn overestimating exposure in peri-urban areas and omitting dense DUAs. Our findings challenge conventional ways of assessing flood risk and emphasize that local knowledge is indispensable in fostering urban resilience.

## Introduction

Urbanization and floods share a wicked interaction: as cities expand towards natural drainage systems, the flood hazard intensifies, exposing more urban areas to risk. This dynamic interaction is context-dependent and particularly complex in Sub-Saharan Africa (SSA), one of the regions most affected by floods globally^1^. The development of SSA cities has been shaped by colonial planning practices, which continue to shape their spatial and social structure of urbanization today. These historical practices concentrated resources and infrastructure in central parts of the cities, thereby serving a limited demographic while marginalizing large segments of the population^2^. As a result, many contemporary SSA cities are characterized by rapid unplanned urbanization, leading to the emergence of Deprived Urban Areas (DUAs)-neighborhoods which face high levels of deprivation, limited infrastructure, tenure insecurity and frequent exposure to hazards^2,3^. Importantly, DUAs are manifested differently across cities and should be treated as context-specific representations of urban deprivation^4^. In SSA, these areas are estimated to house around 60% of the urban population^5^. However, urban planning and disaster risk reduction programmes remain mainly siloed initiatives, focused on formal, planned districts, leaving DUAs outside despite facing great vulnerabilities^6^.

Previous work acknowledged that certain social groups are likely to experience greater flood exposure and risk than others^7–9^. This differential exposure is far from random. In SSA cities, increased flood exposure is often concentrated in areas historically marginalized by planning provision, where settling in floodplains is one of the few options for the urban poor^6^. These areas often coincide with natural drainage networks, are excluded from formal urban development, and are more affordable due to higher flood susceptibility^10,11^. Global assessments estimate that low-income populations are disproportionately affected, especially in the Global South^12^, and that 74 million people are exposed both to flooding and extreme poverty in SSA countries^13^. Future projections also indicate that flood exposure will be concentrated in low-GDP countries, further amplifying impacts in underserved communities^14^. Most flood exposure studies still lack localized assessments capable of distinguishing between DUAs and non-DUAs. Such information, yet scarce, is highly needed in both research and policy, particularly given that exposure and vulnerability assessments are the foundation for equitable disaster risk reduction and adaptation measures^15,16^.

To date, locally accurate flood modeling tools in data-scarce environments remain a challenge. Global flood models, such as GloFAS^17^ or Fathom^18^, though increasingly sophisticated and largely used for global and national assessments, show large inconsistencies at the city scale, with broad modeling assumptions and limited validation in many regions^19,20^. These products also have limited applicability for intra-urban analysis, especially in informal contexts^21^. These models primarily capture large-scale fluvial and coastal flooding, with inadequate resolution of urban pluvial dynamics^22^. Conversely, traditional hydrological models like HEC-RAS^23^ and OpenLISEM^24^ can simulate localized flood dynamics, including pluvial and flash floods. However, their reliance on extensive calibration, high-quality input and technical capacity often renders them unavailable or unaffordable for local authorities in SSA^9^. Consequently, in data-scarce contexts, such methods typically serve as conceptual representations rather than practical instruments for local urban planning.

Compounding this gap is the dominance of top-down flood risk assessment approaches in scientific approaches. The modeling outputs of these approaches are typically assessed through remotely sensed or instrumental data^25^, with little or no involvement of residents, particularly not from those living in deprived settlements^26^. As a result, the rich, experiential knowledge that local communities have about the complex local flood dynamics is commonly ignored. This disconnection between top-down and on-the-ground realities contributes to the systematic overlook of DUAs^14^. In response, this study positions citizen science as a core method to ground our flood model in the lived experience and knowledge of the residents of DUAs.

**Fig. 1:**
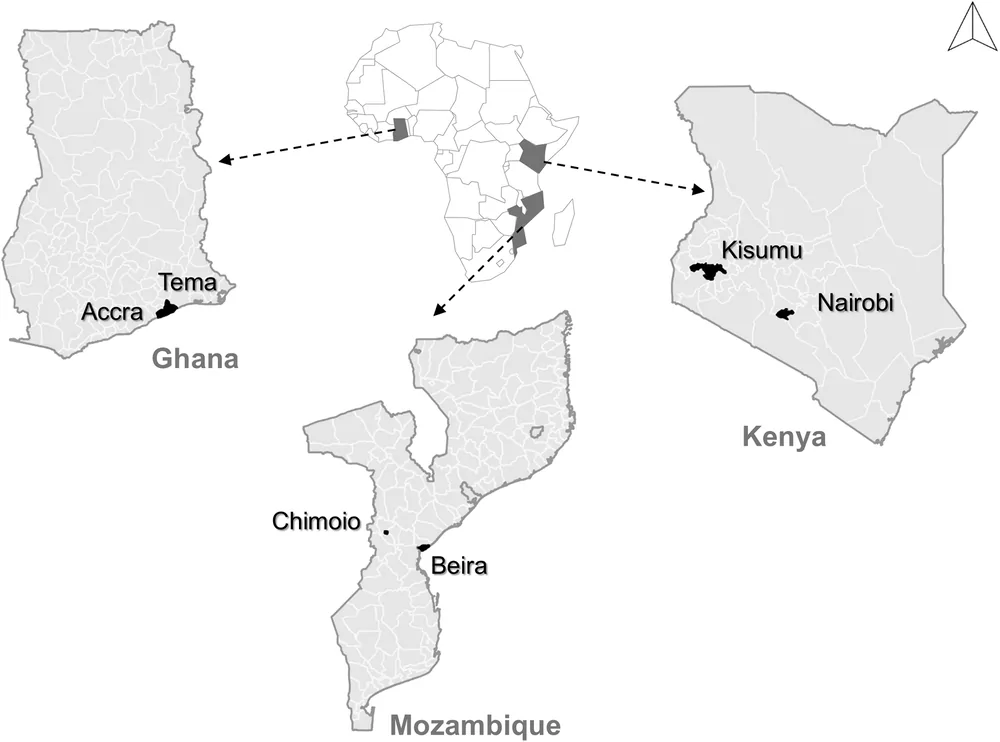
Study areas included in this research. The map shows the geographical distribution of the six Sub-Saharan African study cities included in the analysis: Accra and Tema in Ghana, Nairobi and Kisumu in Kenya, and Beira and Chimoio in Mozambique. The central map indicates the location of the three countries within Africa. Grey national outlines show country boundaries, darker-filled areas indicate the selected countries, and black city markers indicate the approximate location of each study city. The scale is indicative only. Source: FAO GAUL: Global Administrative Unit Layers 2015 through Earth Engine Data Catalog.

Recognizing both the limited evidence of differential flood exposure in SSA-deprived areas and the technical gaps of existing models, we present an alternative approach. Our study integrates a fast simulation flood model, open data and citizen science data to evaluate flood exposure across six SSA cities (Fig. 1) in Kenya (Nairobi and Kisumu), Ghana (Accra and Tema), and Mozambique (Beira and Chimoio). Specifically, we aim to: (1) analyze flood exposure patterns, providing spatial evidence of the alarming flood impacts suffered by deprived urban settlements within and across the cities; (2) assess the findings at the local scale through citizen science, bringing the perspectives of deprived communities into the center of disaster risk assessments. We ask: *How do flood exposure patterns vary across and within SSA cities? And to what extent does the proposed approach capture local realities?* In the next sections, we unpack these questions through spatial, statistical and community-based evidence in an effort to frame flood exposure through the lens of urban deprivation. We adopt an integrated workflow combining: (i) flood hazard modeling, using the FastFlood model, a lightweight steady-state model designed for rapid simulations using open data, offering a performance trade-off in data-scarce urban contexts; (ii) exposure estimation, by intersecting simulated flood depths with built-up density derived from two global datasets; (iii) citizen science assessment, including participatory workshops and community-led mapping surveys in DUAs; and (iv) thematic content analysis of reported flood impacts.

## Results

We examined flood exposure in six SSA cities, specifically in three primary (Nairobi, Accra, Beira) and three secondary cities (Kisumu, Tema, Chimoio). A 50 × 50 m analytical grid was adopted to balance input data resolution, representation of urban morphologies, and computational feasibility, consistent with previous intra-urban studies^27–29^. Flood hazard was first simulated at the basin scale using FastFlood and summarized at three flood depth thresholds (≥1 cm, ≥10 cm, ≥50 cm). To derive flood exposure estimates, the analysis followed two main steps: (i) simulation of flood hazard at basin scale using FastFlood and summarized at three flood depth thresholds (≥1 cm, ≥10 cm, ≥50 cm); (ii) estimation of flood exposure by spatially intersecting simulated flood depths with two built-up density datasets, one derived from the Global Human Settlement Layer (GHSL)^30^ and another from a processed version of Google Open Buildings (GOB)^31^. In this study, exposure is defined as the combination of flood hazard and built-up structures (element at risk). We report both absolute and relative exposure across cities, followed by a comparison between DUAs and non-DUAs. Subsequent sections evaluate the model through citizen validation and assess reported flood impacts through thematic analysis.

### Flood exposure patterns across SSA cities

**Fig. 2:**
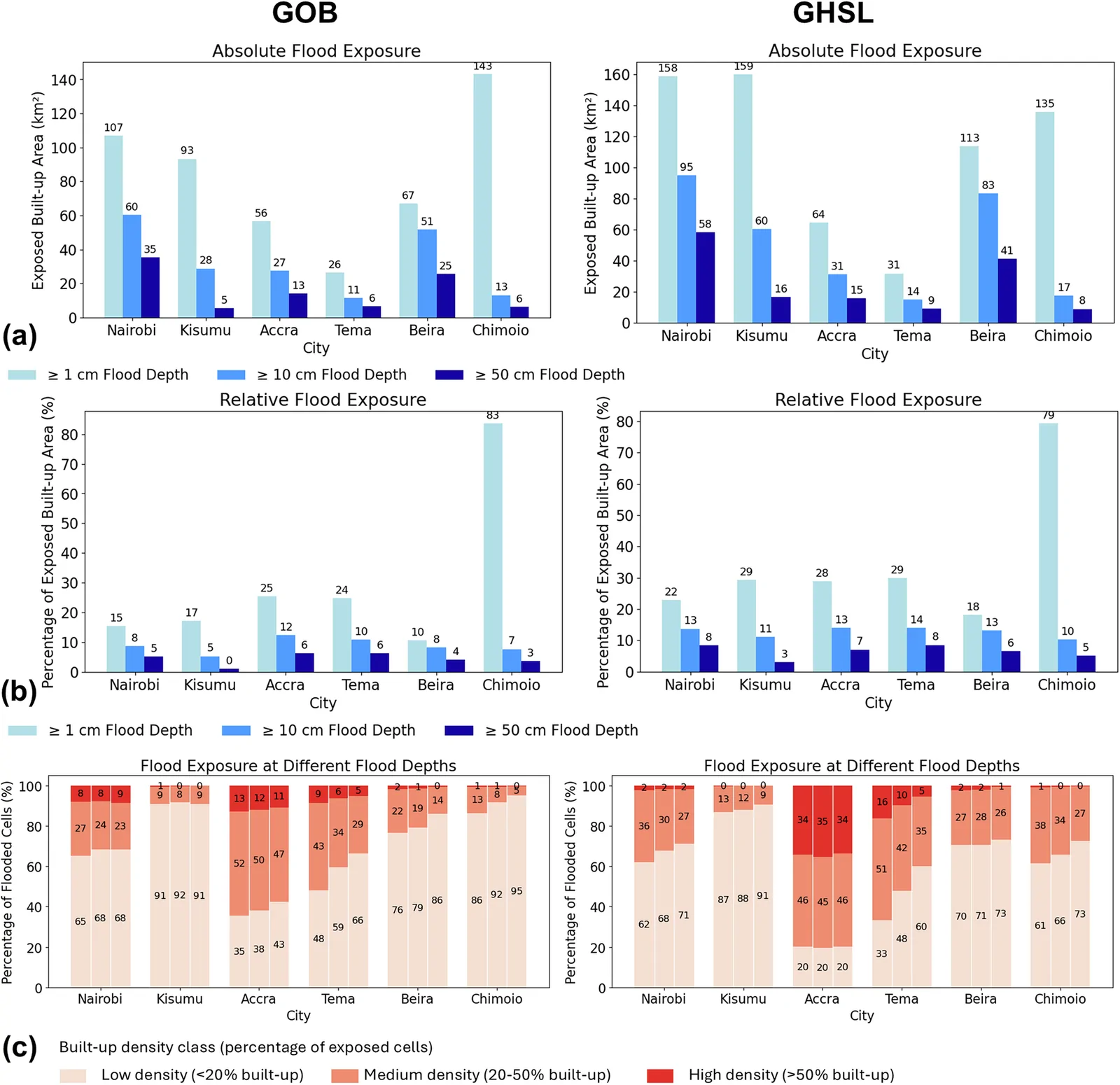
Flood exposure estimates vary across cities, flood-depth thresholds, and built-up datasets. The figure compares flood exposure derived from two built-up datasets: Google Open Buildings (GOB; left panels) and the Global Human Settlement Layer (GHSL; right panels). Flood depths are ordered from left to right in each chart from shallower to deeper flooding: at least 1 cm, at least 10 cm, and at least 50 cm. **a** Absolute flood exposure, expressed as exposed built-up area in square kilometres. **b** Relative flood exposure, expressed as the percentage of total built-up area exposed to flooding. Light-blue bars represent exposure at flood depths of at least 1 cm, medium-blue bars represent exposure at flood depths of at least 10 cm, and dark-blue bars represent exposure at flood depths of at least 50 cm. **c** The distribution of exposed built-up cells by statistically derived built-up density class. Pale peach bars indicate low-density cells, medium red bars indicate medium-density cells, and dark red bars indicate high-density cells.

We observed large differences across cities in the relative flood exposure, and a high variability depending on the used exposure dataset (Fig. 2). Using the GHSL, the proportion of built-up areas exposed to flooding found is 29% in Kisumu and 23% in Nairobi. In contrast, GOB reduced this estimate to 17% and 13%, respectively. While GOB generally reported less exposed areas, due to its exclusion of roads, bare lands and open areas - features often classified as built-up by GHSL- this does not necessarily infer greater accuracy. Road infrastructure, for example, is also an element at risk and can exacerbate flooding by limiting water infiltration. Additionally, the omission of buildings in densely built-up areas in the GOB data^32^ also plays a role in the smaller estimation. Another driver of the differences between GOB and GHSL is the coarser native resolution of GHSL (100 m), which smooths heterogeneous urban fabric, overrepresenting built-up presence in peri-urban areas, while GOB preserves building-level detail prior to aggregation.

Chimoio presents a distinct profile: nearly 80% of its built-up areas are estimated to flood at 1 cm flood depth, declining to 10% at 10 cm. This spatial distribution points to widespread shallow ponding depressions and systematically poor run-off conditions. A notable trend in all cities was the sharp decrease in flood exposure rates between 1 and 50 cm flood heights, suggesting that deeper inundations are more localized. For relative exposure at 50 cm, Nairobi, Beira, and Accra each presented approximately 8% of their urbanized territory exposed to floods. On one hand, this might appear modest, but in absolute values, the size of the built-up areas exposed to floods was substantial, i.e., using GHSL, we calculated flooded areas with 50 cm floods to be 58 km² for Nairobi, 41 km² for Beira and 16 km² for Accra. Considering that large parts of these areas intersect with high-density areas, the estimated impacts on the urban population can have massive consequences. On the other hand, contrary to expectations, absolute exposure was uniformly highest in the primary cities. Aside from Nairobi, Beira (83 km²) and Kisumu (60 km²) registered the highest exposure at the 10 cm flood depth.

The association between exposure and the built-up density categories analysis revealed important patterns. Nairobi, Beira, Accra and Tema (which is also part of Greater Accra) consistently presented higher relative flood exposure (medium and high-density areas that are most flood-prone). The two Ghanaian cities stood out with the largest flood exposure rates. In Accra, the GHSL dataset estimated around 80% of the flooded areas as medium and high built-up density areas across all flood depth thresholds. Tema followed with approximately flooded areas of 52% at 10 cm flood depth and 40% at 50 cm flood depth. Certainly, the urbanization patterns influence the exposed areas, as Accra and Tema portrayed a spread-out urbanization connecting multiple built-up density cores. In contrast, secondary cities with more fragmented urbanization patterns showed a dominant exposure in low-density zones, regardless of the used built-up dataset.

The comparison between the two built-up datasets furthermore revealed consistent spatial classification biases in the representation of built-up density. GHSL generally overestimated the density across all cities, overrepresenting peri-urban and transitional areas, where sparse and heterogeneous land uses are misclassified as high-density fabric. For example, at the 10 cm flood depth threshold, GHSL estimated 35% of Accra’s flooded areas as high density, whereas GOB estimated only 12%. In Nairobi, however, the pattern was reversed; GHSL underestimated dense informal settlements, likely due to its lower sensitivity to complex morphologies^33^.

The general pattern that the percentage of exposed areas shrinks as flood depth increases emerged, but was less pronounced than expected. The data suggested that the same neighborhoods are repeatedly affected, regardless of the flood depth thresholds (Supplementary Fig. 1). These persistent exposure patterns were especially clear in high-density areas, which might be connected to the highly dense DUAs, the limited drainage capacity from imperviousness, inadequate infrastructure, and management capacity^21^.

### Differential flood exposure: deprived vs non-deprived areas

Analyzing the intra-urban patterns by overlaying flood exposure with the DUA’s boundaries, this study highlights sharp disparities: our estimates showed that in high- and medium-density zones, DUAs are up to 47% more exposed to floods above 10 cm when compared to non-DUAs. When considering only high-density zones, this ratio rises to 60%. However, we report 47% as a more representative comparison across cities with different urban forms. In secondary cities, few areas meet the high-density threshold, yet many medium-density DUAs are functionally equivalent to high-density contexts in primary cities. Including medium-density areas provided a more consistent measure of intra-urban assessment, avoiding underrepresenting flood exposure in smaller cities.

**Fig. 3:**
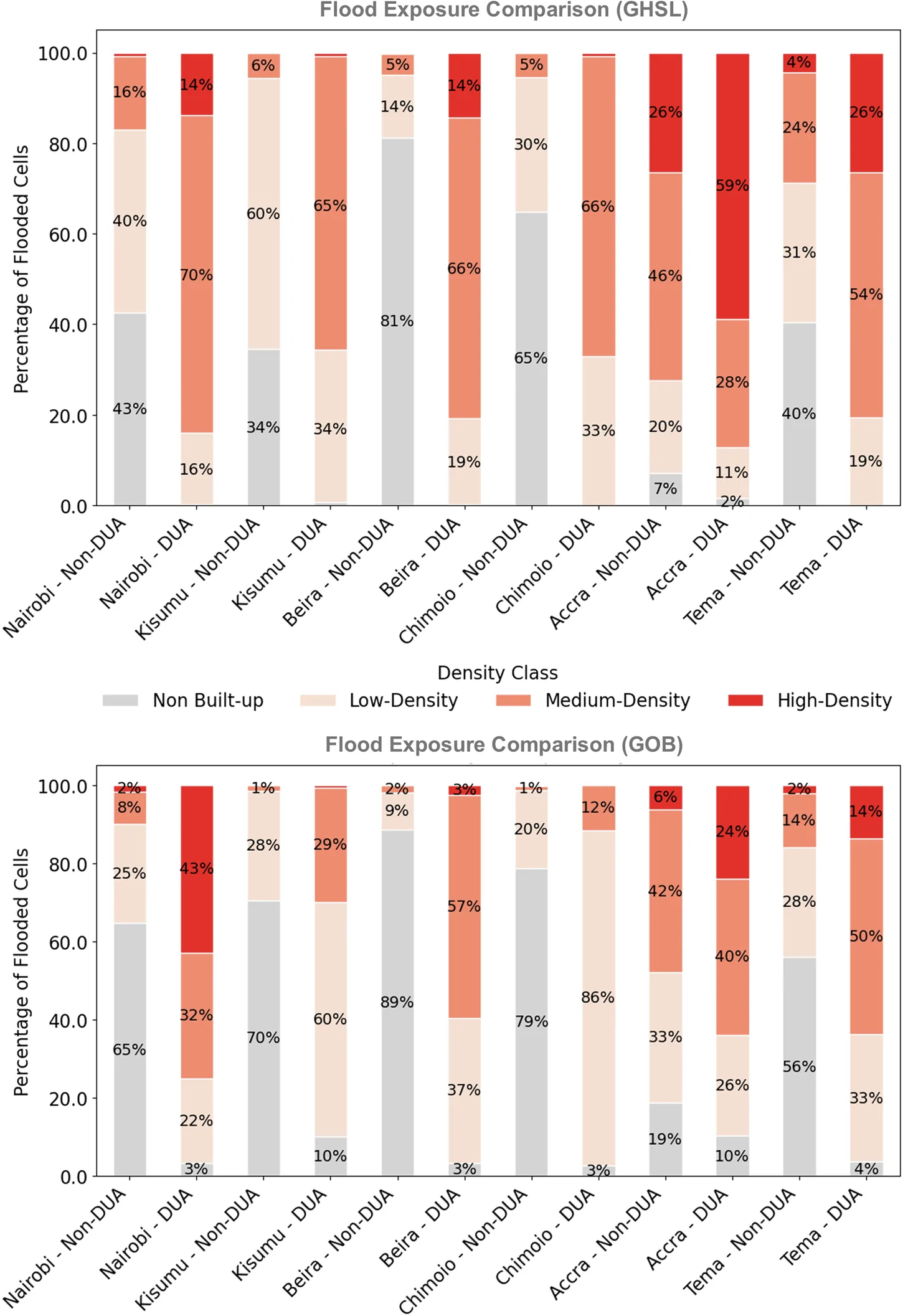
Flood exposure comparison between deprived (DUA) and non-deprived (Non-DUA) areas. The upper panel shows estimates derived from the Global Human Settlement Layer (GHSL), and the lower panel shows estimates derived from Google Open Buildings (GOB). The *y*-axis shows the percentage of flooded cells, and the *x*-axis compares DUA and Non-DUA areas for Nairobi, Kisumu, Beira, Chimoio, Accra, and Tema. Grey segments indicate non-built-up cells, pale peach segments indicate low-density built-up cells, orange segments indicate medium-density built-up cells, and red segments indicate high-density built-up cells.

The degree of exposure inequity varied considerably across the cities. Nairobi exhibited the highest overexposure, with DUAs being two times more exposed than non-DUAs, followed by Kisumu (52%), Accra (46%), and Beira (17%). In contrast, Chimoio and Tema showed near parity in exposure levels between DUAs and non-DUAs. This might indicate that, in secondary cities, limited overall investment in infrastructure and disaster preparedness leaves the entire city exposed, narrowing the intra-urban differences^2^. Figure 3 presents the proportion of flooded areas in each city and underscores the divergences between DUAs and non-DUAs: flood-prone DUAs are consistently characterized by higher built-up densities. In most cities, flooded non-DUA zones coincide with non-built-up land, whereas flooded DUAs are predominantly located in moderately and highly built-up environments. This pattern is evident across both built-up datasets - GOB and GHSL - though GHSL consistently provides larger estimates in both DUA and non-DUAs, with the exception of the city of Nairobi. This outlier is likely due to the differences in dataset completeness: as a primary megacity and pilot site for the GOB initiative, Nairobi benefits from more detailed and accurate coverage. Compared to other building footprint datasets, GOB reports the smallest average building area per structure, indicating greater sensitivity to detecting smaller structures, which is particularly relevant for high-density consolidated settlements such as the ones in Nairobi^34^. By contrast, previous work has proven that GHSL tends to underestimate highly built-up areas and overestimate peri-urban areas in secondary SSA cities^35^.

**Fig. 4:**
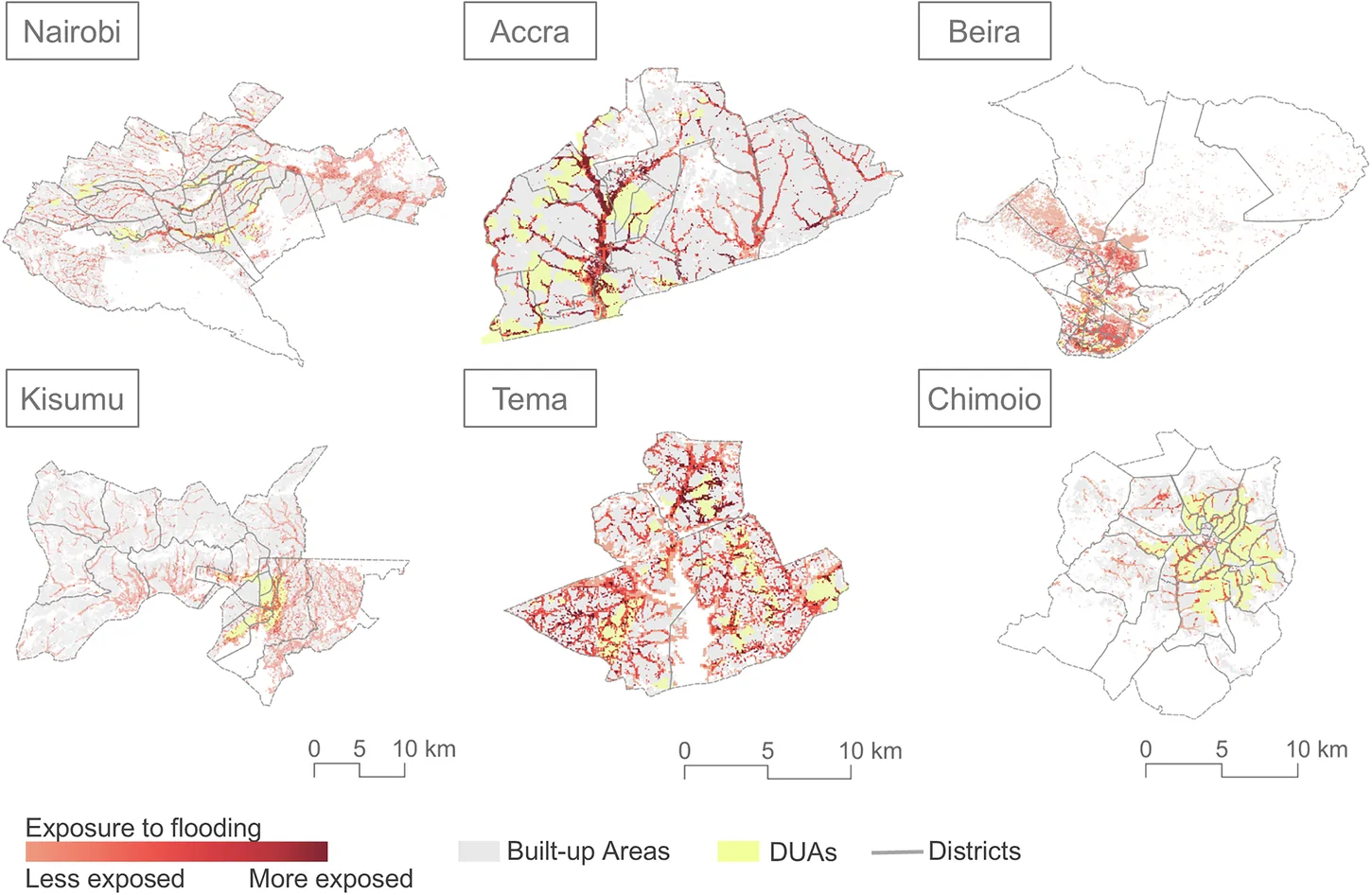
Flood exposure map across cities. The six city maps show flood exposure estimates derived from the Global Human Settlement Layer (GHSL) and overlaid with deprived urban area (DUA) boundaries. The red exposure gradient represents flood exposure, with lighter red indicating lower exposure and darker red indicating higher exposure. Grey areas represent built-up areas and yellow areas represent DUAs.

The mapped results indicate that flood exposure is concentrated in specific areas, where both geography and urbanization play a critical role. Across all cities, highly exposed areas tend to cluster close to or along riverine areas, confluences and low-lying zones, frequently home to DUA populations (Fig. 4).

In Nairobi, the most pronounced case, highly dense and deprived settlements situated along major tributaries show consistently higher exposure, reflecting long-standing urban planning decisions. In Kisumu, the eastern side of the city is considerably more exposed, with the largest ratio of high flood exposure for the districts of Manyatta B, Nyalenda and Kolwa Central, near the Nyamasaria River. In Accra, exposure is concentrated along the Odaw River, where many industrial areas and DUAs encroach on originally demarcated buffer zones, but now occupied due to land scarcity and weak enforcement. In Tema, the Ashaiman district, close to the Ashaiman Dam, notably exhibits the highest exposure levels, reflecting unplanned expansion into areas originally reserved for farming, unsuited for residential use, and deprived of adequate infrastructure and services. In Beira, many DUAs surrounding the Central Business District (CBD) are estimated as highly exposed, particularly in districts such as Ndunda, while redeveloped coastal areas within the CBD show minimal to no exposure. The same was seen in the central part of Chimoio, where flood exposure is largely riverine and spatially concentrated south of the CBD, most prominent in the districts of 25 de Setembro and Centro Hípico. Together, these patterns illustrate how flood exposure is shaped both by geography and uneven urban development.

**Fig. 5:**
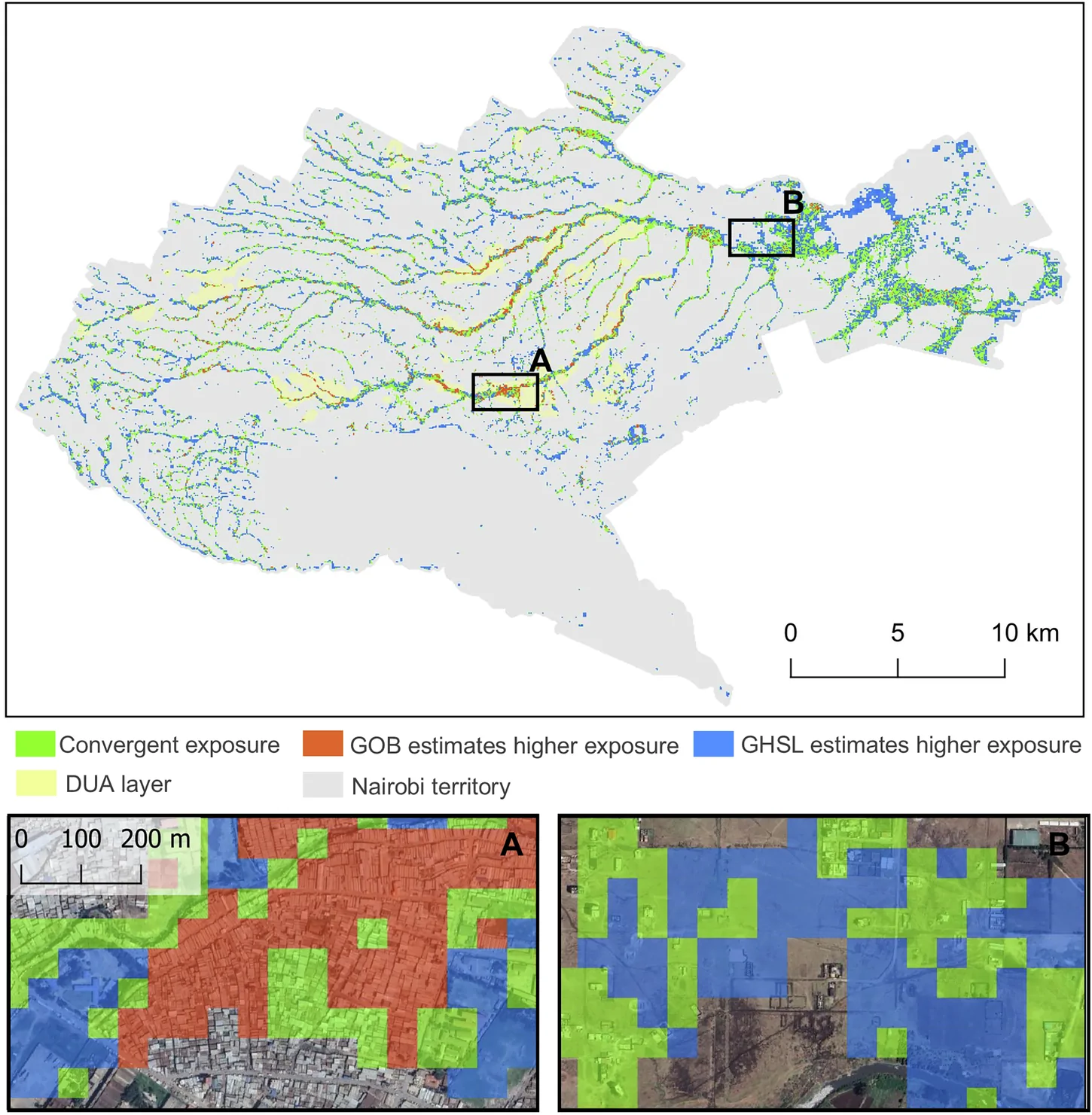
Agreement between GOB- and GHSL. The main map shows the level of agreement between flood exposure estimates derived from Google Open Buildings (GOB) and the Global Human Settlement Layer (GHSL) in Nairobi. Green cells indicate convergent exposure estimates, orange cells indicate areas where GOB estimates higher exposure, and blue cells indicate areas where GHSL estimates higher exposure. Black boxes identify two zoom-in areas. **A** Mukuru; **B** Ngundu.

A closer inspection of exposure patterns reveals systematic differences between estimates derived from GOB and GHSL, which are consistent across the study areas. Figure 5 presents Nairobi as an illustrative example, as it exhibits the strongest divergence between datasets and therefore highlights how dataset choice matters. Large portions of the city show convergence between GHSL and GOB, particularly in medium-density areas where both datasets consistently represent built-up areas. In contrast, divergence is evident in dense informal settlements along the tributaries, such as Mukuru settlement (Fig. 5A), where GOB classifies cells as highly exposed while GHSL assigns lower density classes, reflecting GHSL tendency to smooth heterogeneous urban fabric and underrepresent informal areas. Conversely, in early-stage development and peri-urban areas, GHSL estimates are higher when GOB does not even assign them as built-up (Fig. 5B).

Since our analysis did not differentiate among types of elements at risk, such as roads, buildings, open areas, higher built-up density is directly interpreted as higher potential impact. Yet this assumption can mislead, because the two datasets have different assumptions. GHSL considers other elements at risk as built-up, while GOB only considers buildings. Given that damage to roads and open areas as less severe than damage to buildings (often life-threatening), the estimates from GOB seemed more aligned with ground realities. That said, the pattern of underestimation by GOB also downplays exposure in suburban or early-stage development zones and overestimates exposure in highly dense areas. Therefore, at first sight, the GHSL’s broader completeness and higher estimates seem to be the best alternative at the city scale, but at the community scale, the GOB can provide a more spatially accurate representation of differential exposure, especially in dense DUAs.

### Citizen Science insights – participatory evaluation of flood patterns

Given the stark disparities in exposure, we assessed whether the model accurately captured these patterns, particularly in the most exposed DUAs, where systematic assessments are absent, yet flood impacts are expected to be most severe^13,21^. To do this, we integrated the flood model with two complementary citizen science approaches: local workshops and community-led mapping surveys. Across the six cities, in total 21 workshops were conducted, engaging residents, local leaders, NGOs, city authorities and academic partners (*n* = 255). With support of local partners, in 16 communities, participants captured contrasting urban morphologies, stages of development and hydrological settings. In parallel, the residents co-developed a strategy and mapped flood observation points using Google My Maps, resulting in 310 entries after quality control (Supplementary Figs. 8 and 9).

These participatory methods provided a range of findings. The workshops with city planners generally found that the FastFlood model^36^ provided valuable estimates (in terms of flood extent and depth), despite limited input data availability and computation requirements. Participants agreed the model had an underestimation tendency in DUAs, indicating these areas as highly exposed, meanwhile suggesting that the model overestimated floods in formal areas, where improvement and investment in flood mitigation had taken place.

**Fig. 6:**
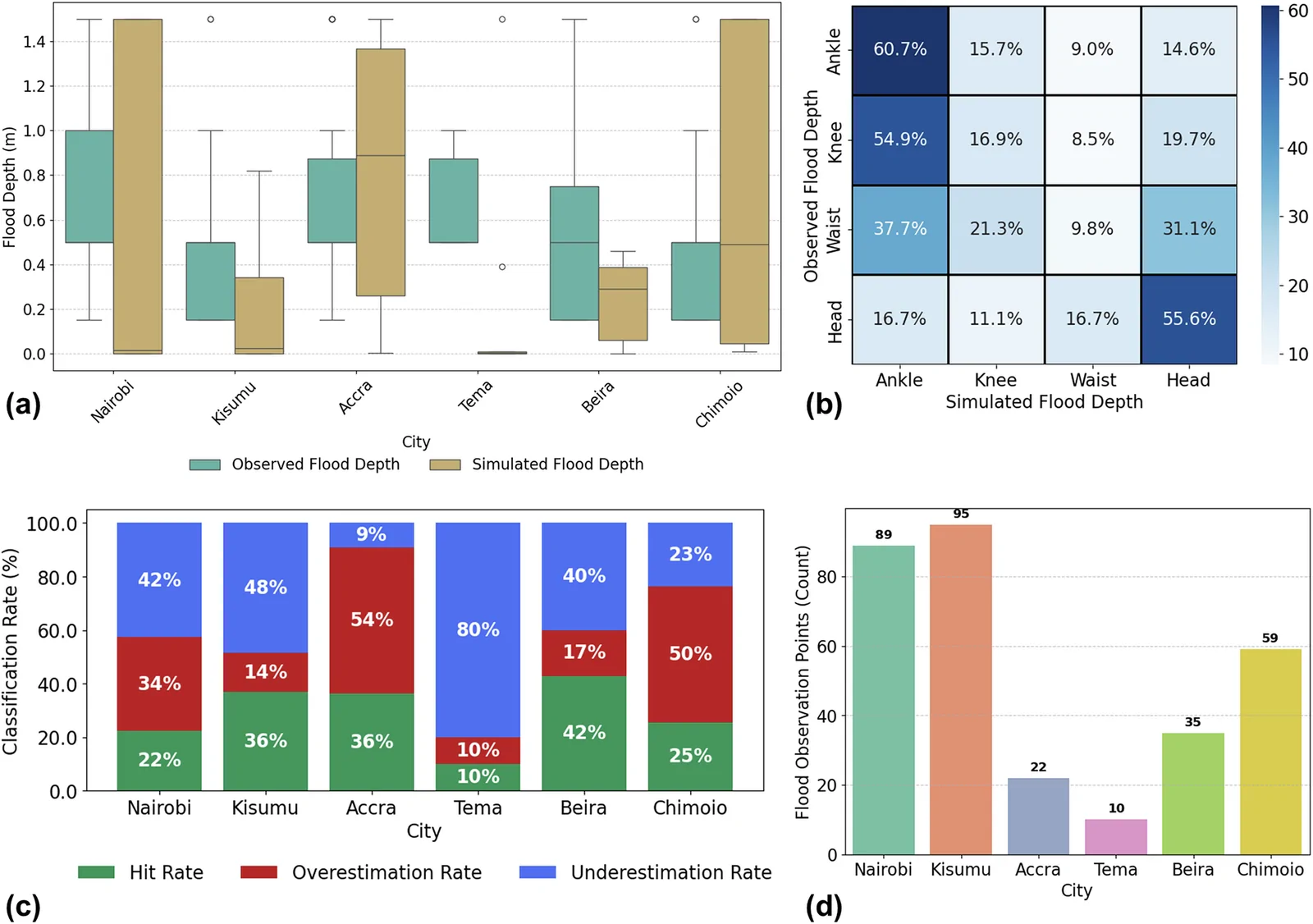
Citizen-science flood observations show that FastFlood model performance varies by city and flood-depth class. **a** Boxplots of observed and simulated flood depths across cities, capped at 1.5 m for comparability. Green boxplots represent observed flood depth, and mustard boxplots represent simulated flood depth. **b** A confusion matrix comparing observed flood-depth classes with simulated flood-depth classes. Rows indicate observed classes, columns indicate simulated classes, and darker blue cells indicate higher percentages of observations in each class combination. **c** Stacked bars of the hit-and-miss evaluation by city. Green segments indicate hit rate, red segments indicate overestimation rate, and blue segments indicate underestimation rate. **d** The number of flood observation points per city, with one colored bar for each city.

The participatory survey highlighted the underestimation of floods in DUAs. Results showed that 60% of ankle-level and 50% of the head-level floods were correctly predicted (Fig. 6). However, the model often simulated ankle-depth floods where residents reported knee or waist-level floods. The findings reflect the model’s tendency to underestimate or even omit artificial flooding, caused by human-induced factors such as drainage issues, blocked waterways or overall poor infrastructure.

Comparing the data distribution across cities, we found that underestimation was also city dependent (Fig. 6). In Nairobi, simulated flood depths varied highly, with localized extremes and considerably lower medians, indicating systematic underestimation. Contributing factors might include: (1) the Digital Elevation Model (DEM) limitations, e.g., vertical accuracy, even though proven to be better than ASTER, ALOS, and NASADEM in SSA countries^37^; (2) the steep topography of the city and the consequent increasing runoff estimates^38^, (3) and the deviations of the streams at the local level. The latter factor was largely brought up during the local workshops, since the model mistakenly captures points inside the streamlines (following the DEM), while these have been deviated from the ground. Statistical inspection showed that half of the simulated values for ankle-level floods reached only 1 cm, confirming the structural underestimation by the model. Additionally, from the workshops we learned that the modeled extent focuses on the main tributaries, often neglecting secondary channels that also contribute significantly to natural drainage and flood impacts. This effect is likely related to the DEM resolution and the hydraulic assumptions of the model, prioritizing dominant flow paths while underrepresenting small or informal drainage structures that play a big role in flood dynamics in DUAs.

Kisumu and Beira followed the same underestimated orientation. In Kisumu, the model failed to capture backflow from the lake, while in Beira, although the overall agreement was high, the flood extent was still underestimated in the Munhava settlement, which was subject to artificial flooding. Conversely, Accra and Chimoio presented overestimation patterns. Both had wide interquartile ranges similar to Nairobi, but with higher medians. In Accra, overestimation was mostly concentrated in the Old Fadama area that has gone through recent development interventions, not captured by the model. In Chimoio, the observed overestimation likely results from a combination of the DEM coarse resolution, local deviations of drainage pathways and field survey uncertainties inherent in complex dense urban environments, which affect the validation through positional errors and reporting variability.

Tema had the highest underestimation rate, partially related to the artificial floods, frequently reported by the residents in the workshops, but also influenced by the limited number of surveyed points - the smallest among all cities (Fig. 6). Accra and Tema together had fewer than 50 surveyed points, whereas Kisumu alone had almost 100. As we co-developed the survey process with the residents, we asked them to map observed flood events representing the flood situation in their communities until they were satisfied. This process provided flexibility in terms of hours and points surveyed, but introduced variability in coverage. The level of commitment and support of the local leaders and the digital literacy of the participants certainly influenced the variability in the data.

This locally derived flood data was treated as experiential evidence to evaluate the model and identify systemic biases. Validation relied on triangulation across sources, including agreement between multiple workshop groups and comparison with modeled flood depths.

### Content analysis in deprived areas – what residents say about flooding

Up until here, the analysis shows that the model captures flood patterns well at the city scale but significantly underestimates flood intensity in DUAs. This is problematic. However, further inconsistencies emerged when comparing the observed flood depths with the reported impacts. To explore this further, we conducted a thematic analysis of 310 community-reported flood points, identifying nine overarching impact categories which align with the sectoral effects from the Sendai Framework targets^39^ (see details on “Methods”).

**Fig. 7:**
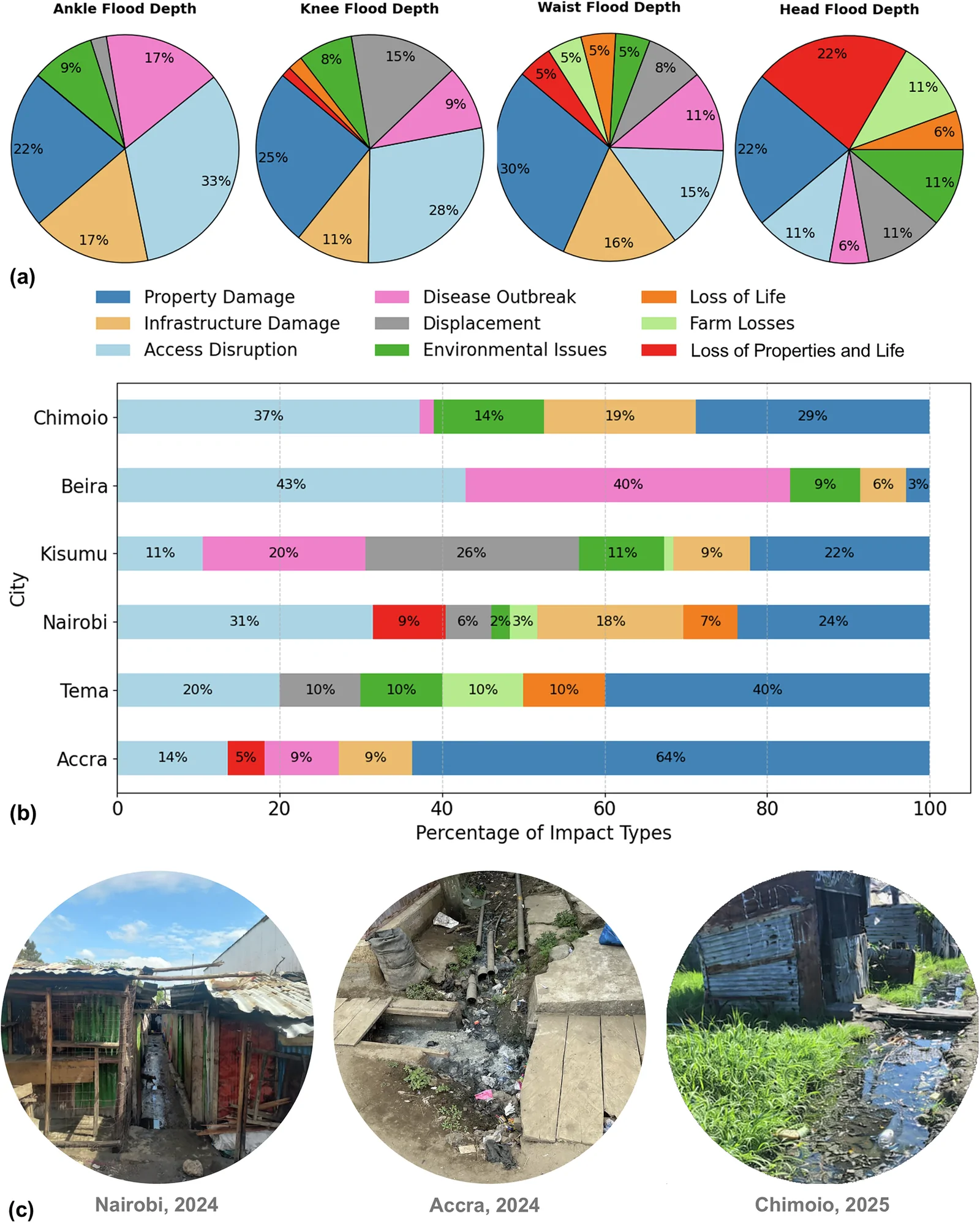
Community-reported flood impacts. **a** Pie charts of impact themes across four observed flood-depth categories: ankle, knee, waist, and head level. **b** The percentage distribution of impact themes across cities. The *x*-axis shows the percentage of impact types, and the *y*-axis lists the cities. Colors identify impact themes. **c** Fieldwork photographs from three deprived urban areas: Nairobi in 2024, Accra in 2024, and Chimoio in 2025.

The distribution of the impacts across the observed flood depth categories revealed that shallow floods—up to ankle height—are associated with substantial impacts. 33% of the reported impacts at ankle-level floods were linked to access disruption issues, while over 50% of the impacts reported property damage, disease outbreak and infrastructure damage (Fig. 7a). These findings challenge the prevailing assumptions that flood depths below 15 cm are non-hazardous, as if shallow flooding only impairs mobility of daily activities. In contrast, lived experiences in the DUAs indicate that shallow flooding can result in serious impacts, extending far beyond impaired mobility of daily activities^26^.

City-level patterns further reinforced these insights (Fig. 7b). Disease outbreaks, notably prominent in Beira, Kisumu and Accra, were concentrated in low-lying settlements near large water bodies such as the Indian Ocean and Lake Victoria. These locations are flood-prone and suffer from poor drainage systems, and consequently, stagnant water, increasing vulnerability to health-related impacts. The larger economically central cities—Nairobi, Tema and Accra—showed correspondence regarding high-severity impacts, such as property destruction and loss of life, suggesting an association between the scale of urbanization and the concentration of flood risks. Another pattern emerged from the secondary cities— Chimoio, Kisumu and Tema—with larger shares of impacts linked to environmental issues (soil erosion, environmental contamination). Workshop discussions revealed a regular practice of landlords in DUAs, where they used floodwaters to decongest the latrines, intentionally worsening water and soil pollution. Our field observations confirmed these reports, revealing how poor environmental and housing infrastructure can make even shallow floods highly disruptive and damaging (Fig. 7c). An additional and unexpected result was that, in Accra and Beira, displacement was not reported as an impact. From the workshops, we learned that it is partially true, as in the case of the destruction of property, the affected residents opt to reside with friends and relatives close by, while they will rebuild their homes on the original spot after the rainy season.

## Discussion

Recent global studies have demonstrated that flood exposure disproportionately affects low-income populations worldwide, particularly in the Global South^12,13^. Building on this work, our contribution lies in systematically centering DUAs within a comparative analysis in Sub-Saharan Africa, explicitly quantifying differential exposure between DUAs and non-DUAs, and grounding modeling results into citizen science approaches. Across cities of varying sizes and morphological and geographical contexts, our results consistently show higher flood exposure in DUAs, especially at medium and high built-up densities. In Nairobi, the most extreme case, DUAs were found to be over 200% more exposed than non-DUAs. Importantly, these disparities are evident not only in primary cities like Nairobi and Accra, but also in secondary ones such as Kisumu and Beira, highlighting the systemic nature of these inequalities. Given projections of increasing flood exposure in low-income urban areas^14^ and limited relocation options for many residents^40^, these findings underscore an even more pressing and complex challenge.

To address technical and data constraints common in many LMICs—including the absence of city-scale flood risk expertise^2,20^ and by the high costs of commercial tools^9^ - our methodology adopted a lightweight flood modeling approach combined with open datasets. Rather than aiming for event-based, physically detailed simulations, the approach prioritizes computational efficiency, transparency of assumptions and comparability across cities. The FastFlood approach enables the identification of intra-urban exposure patterns that are often smoothed in national or global flood products, particularly in complex urban contexts. Albeit not the first to map flood exposure in SSA cities using available remote sensing data^9,22^, the comparative focus on DUAs provides new insights into how exposure inequalities manifest across different urban contexts.

In parallel, uncertainty in exposure estimates was explored using two built-up datasets, GOB and GHSL. The systematic overestimation of exposure from GHSL - especially in peri-urban and transitional zones- highlights how exposure estimates are shaped by the structure and assumptions of the “elements-at-risk” datasets. In contrast, the building-level source from GOB captures dense and fragmented urban fabrics within DUAs. While this study focuses on built-up structures as a proxy for exposure, future work could refine these estimates by incorporating building typologies, potentially drawing on Volunteered Geographic Information such as OpenStreetMap tags^41–43^. Given the influence of input data on flood risk assessments and subsequent policy decisions, continued efforts towards data validation and triangulation remain critical.

Citizen science (CS) played a central role in addressing methodological blind spots inherent in flood modeling in deprived contexts. Beyond serving as a validation step, participatory mapping and workshops generated practical insights into flood impacts and revealed systemic mismatches between modeled flood depth and the lived experience^44,45^. In particular, residents consistently reported severe disruptions such as disease outbreaks, damage to properties and soil contamination, under shallow flooding conditions (below 15 cm). These findings challenge commonly used flood depth thresholds in flood assessments and provide actionable insights into flood impacts. Previous work indicated that the most densely built-up areas are affected by low-frequency and high-magnitude flood events^46^. Yet, our results show that, in deprived contexts, low-magnitude high-frequency flooding can pose compounding impacts on livelihoods, infrastructure, and public health.

The integration of CS also carries broader methodological and epidemiological implications. Community engagement helped contextual model outputs by revealing local processes—such as informal drainage modifications and maintenance failures- that are not explicitly represented in most flood models. More broadly, this approach responds to longstanding critiques of technocratic urban science by foregrounding local knowledge in contexts where residents are often excluded from data production and decision-making^2^. For many, participation in science - and politics - must be negotiated alongside daily struggles for food, housing, and safety^45^. Certainly, the legacy of colonialism still reverberates^10^, and in this context, urban science has been shaped by the values, needs and resources of the Global North^47,48^, highlighting a deep asymmetry in how knowledge is produced, valued and mobilized globally. This can have serious consequences in decision-making by reinforcing existing inequalities in the flood risk assessments we attempt to carry out today. As technology continues to shape the urban research agenda, we must ensure it does not deepen existing divides^10^. Importantly, the CS framework adopted here relies on open, lightweight digital tools and locally facilitated workshops that can be easily adapted without advanced technical capacity by local stakeholders in other data-scarce cities. This supports iterative, locally grounded flood risk assessment. We argue that participatory validation processes should not be treated as one-off exercises, but should rather be integrated into flood risk policies for more realistic models and more just outcomes^25,49–51^.

Our methodology is subject to important considerations. First, the flood model represents a static state with simplified hydrological assumptions that limit its ability to capture processes such as lake backflow in Kisumu, coastal surges in Accra, or artificial flood factors as blocked drainage, which are particularly relevant in DUAs^46,52^. This information is not easily represented in hydrodynamic flood models^53^, especially considering the informality of the systems in place. Second, data uncertainties arise from: (i) incomplete local precipitation data, where we may have missed short-duration rainstorms that can significantly affect urban flooding^54^; (ii) differences in spatial resolution and modeling assumptions. Higher flood exposure estimates from the GHSL layer relate to the inclusion of other built-up structures aside from buildings^35^ and its coarser native spatial resolution that smooths out fine-scale urban heterogeneity^55^. Unfortunately, these uncertainties are difficult to quantify in the absence of high-frequency ground truth data^4^; (iii) temporal and conceptual inconsistencies. Different temporal coverage of the datasets may affect the absolute flood exposure estimates^55^. Yet, the comparative exposure patterns observed across cities are more robust to short-term (less than 5 years) changes, unless going for extreme transformation like a massive post-disaster destruction^56^. These temporal mismatches can partly explain disagreements between simulated flood patterns and community-reported impacts. In particular, the DUA boundaries, even though locally validated, are derived from different data sources and mapping approaches, and are therefore subject to omissions and influenced by the subjective confidence of mappers (in cases of delineation through visual interpretation). To account for these uncertainties, the analysis is conducted at an aggregated level, where main findings—based on comparison between DUAs and non-DUAs- are less sensitive to boundary issues. Third, the CS component relied on non-random participation (with participant compensation), which may cause selection biases. Additionally, uneven observation density across cities, differences in local engagement, digital access, and time availability influenced data collection. Yet, even with these uncertainties influencing statistical representativeness, the approach provided contextual accuracy and the inclusion of lived experiences typically absent from formal assessments.

Overall, our findings demonstrate that flood exposure patterns are unevenly distributed across and within the six SSA cities and consistently concentrated in DUAs, where it is often underestimated by conventional assessments. By combining light-weight modeling, open datasets and participation mapping, this study offers an operational and replicable approach that is grounded in local realities for identifying differential exposure in data-scarce urban contexts. While subject to important limitations, the approach provides a foundation for more equitable flood exposure analysis and aligns with global agendas on sustainable cities (SDG 11) and climate action (SDG 13), insisting on just outcomes, but also methods and processes. Future research should build on this work by integrating explicit vulnerability indicators—such as housing quality, access to infrastructure and adaptive capacity^21^—and temporal dynamics to move toward more comprehensive and just flood risk assessments.

## Methods

### Case studies

The study focuses on six cities across three SSA countries (Kenya, Ghana and Mozambique) that exhibit high human vulnerability to climate change yet face significant data scarcity^16^. These cities span coastal, riverine, and inland flood contexts (Fig. 1). Three primary cities (Nairobi, Accra and Beira) and three secondary cities (Kisumu, Tema and Chimoio) were selected based on three criteria: (i) the increasing threat of urban flooding, documented through recent severe flood events; (ii) representation of diverse urban morphologies; and (iii) established collaboration with local partners.

To contextualize the risk profiles of each city, we conducted a review of the most severe flood events reported over the past decade (Supplementary Table 1). Sources included scientific publications, websites, and government records, which were cross-referenced with the Dartmouth Flood Observatory^57^ database for validation. This review highlighted the diversity of flood dynamics and the unique urban conditions across the case studies.

In Ghana, Accra and Tema share a coastal location and the same metropolitan governance but considerably differ in urban form and density^58^. In Kenya, the 4 million residents of Nairobi regularly experience intense flash floods, driven by rapid run-off in steep urbanized catchments. Conversely, Kisumu, a secondary mid-sized city on the shores of Lake Victoria, faces compounded risks from the lake backflow and riverine flooding, particularly during the rainy season^59^. In Mozambique, Beira, located at the mouth of the Pungwe river delta, is often exposed to cyclones, while Chimoio, with half of the population of Beira, is located inland and surrounded by river valleys, illustrating the challenges of flood risk in secondary cities^60^.

Following city selection, DUAs were identified as focal sites for detailed analysis within each city based on their hydrological susceptibility and local contextual relevance. Areas prone to surface water accumulation were first screened using Stream Power Index computation, indicating critical points likely to experience concentrated run-off and flooding (Supplementary Table 6). DUAs located within or adjacent to these zones were subsequently selected in collaboration with local partners, considering diverse urban morphologies and development stages.

### Data

In this study, we used globally available datasets, open repositories and citizen-generated data. Table 1 provides an overview of each dataset, its respective properties and sources. Although population exposure is central to flood assessments, we selected two built-up density layers to represent physical elements at risk. Gridded population products rely on inputs that are highly uncertain in informal complex urban areas, leading to substantial misrepresentation of population distribution in DUAs^35,55^.

**Table 1:** Overview of datasets used in this study

Data	Dataset name	Properties	Source
City extent	Global Administrative Areas (GADM)	Polygon vector 2022	https://gadm.org/data.html
Basin extent	WWF HydroSHEDS	Polygon vector 2000	https://earthengine.google.com/
Rainfall*	Climate Hazards Group InfraRed Precipitation with Station Data (CHIRPS)	Raster (0.05° ~ 5 km) Daily	https://earthengine.google.com/
Rainfall*	Global Satellite Mapping of Precipitation (GSMaP)	Raster (0.1° ~ 11 km) Hourly	https://earthengine.google.com/
Rainfall	Trans-African Hydro-Meteorological Observatory (TAHMO)	Tabular (aspatial) 2019–2024	https://tahmo.org/climate-data/
Terrain*	Shuttle Radar Topography Mission (SRTM)	Raster (30 m) 2014	FastFlood Repository
Terrain	ESA Copernicus G-LO	Raster (30 m) 2015	FastFlood Repository
Land cover	ESA Copernicus WorldCover	Raster (10 m) 2020	FastFlood Repository
Soil moisture	SOILGRIDS	Raster (250 m) 2017	FastFlood Repository
Built-up density	Google Open Buildings (GOB)	Polygon vector 2021 (aggregated to 50 × 50 m grid)	https://earthengine.google.com/
Built-up density	Global Human Settlement Layer (GHSL)	Raster (100 m) 2025	https://human-settlement.emergency.copernicus.eu/download.php
Deprived urban areas	DUAs/Slums	Polygon vector (Various years)	Compiled from multiple sources**
Citizen-generated data	Flood Observation Points	Point vector 2024–2025	Google My Maps Survey

The table summarizes the datasets used for flood modelling, exposure analysis, deprived urban area delineation, and validation. Columns report the data category, dataset name, spatial or tabular properties, temporal coverage, and source. Single asterisks identify datasets initially explored but excluded from further analysis. Double asterisks identify datasets available only upon request because of privacy considerations. See Data Availability for access and further detail.

The datasets used in this analysis span different years, reflecting the realities of working with open data in data-scarce contexts. While these temporal mismatches introduce uncertainty—particularly in rapidly evolving DUAs—we assume that the main urban and natural landscape patterns remain sufficiently stable for comparative analysis. DUAs were delineated from multiple sources, using previous research (Table 2), resulting in mixed conceptual framings and mapping approaches across cities. We acknowledge these variations may introduce spatial inconsistencies^61^, but we used existing delineations to preserve contextual relevance since high-quality locally grounded datasets were already available.

**Table 2:** Overview of DUA datasets

City	Conceptual framing	Mapping approach	Year	Validation	Source
Nairobi	Observable deprivation features + contextual knowledge	Supervised classification + in situ mapping	2021	Local stakeholder validation	SLUMAP project
Kisumu	Observable deprivation features + contextual knowledge	Supervised classification + in situ mapping	2021	Local stakeholder validation	SLUMAP project
Accra	Observable features + contextual knowledge	Supervised classification + in situ mapping	2020	Local stakeholder validation	SLUMAP project
Tema	Observable features + contextual knowledge	Visual interpretation of EO imagery	2025	Expert-led local validation	Space4All project
Beira	Observable features + contextual knowledge	Visual interpretation of EO imagery	2025	Expert-led local validation	Space4All project
Chimoio	Observable features + contextual knowledge	Visual interpretation of EO imagery	2025	Expert-led local validation	Space4All project

The table summarizes the deprived urban area (DUA) datasets used. Columns report the city, conceptual framing, mapping approach, year, validation method, and source. Sources: SLUMAP project https://slumap.ulb.be/ and SPACE4ALL project https://www.itc.nl/space4all/.

### Methodological approach

To achieve reliable flood hazard and exposure estimates this study consists of four main steps: (i) the development of a flood hazard model using FastFlood model and open data sources; (ii) the mapping and estimation of flood exposure using GOB and GHSL; (iii) the local validation with citizen science approaches; (iv) the thematic analysis of flood impacts reported by the residents from deprived areas.

Flood modeling was performed with FastFlood tool, a web-based application that uses steady-state assumptions—and further mathematical corrections—to enable high-speed simulation and minimal parameterization^36^. The flood simulations produced in this study represent static flood hazard conditions derived from historical rainfall analysis, using high precipitation peaks identified from local gauge data and cross-validated with reported flood events and literature. The modeling framework primarily focuses on pluvial and fluvial flooding processes driven by rainfall-runoff dynamics. Other processes, such as lake backflow (relevant in Kisumu) and coastal surge (relevant in Accra, Tema and Beira), are not explicitly represented. Flood exposure estimates in these cities are likely higher on the ground, as the model will only reflect the pluvial/fluvial component of flooding.

Catchment areas were delineated using a HydroSHEDS dataset, and aligned with GADM administrative boundaries to ensure complete spatial coverage (details in Supplementary Tables 4 and 5). The catchment extents were chosen to balance hydrological accuracy and computational feasibility.

Rainfall analysis involved a series of tests for the pilot city. First, satellite-derived datasets (CHIRPS and GSMaP) were tested without downscaling techniques, but results were found too coarse for city-scale application, leading to misconceptions of the precipitation distribution^7,62^. We therefore adopted TAHMO precipitation data from local gauge stations, which, despite the shorter time series (from 2019 to 2024), offered hourly resolution and reasonable coverage across cities, defined here as at least two operational gauge stations within or adjacent to the urban boundary. Hourly precipitation records were aggregated (daily and yearly) and analyzed to identify high-intensity rainfall peaks and assess their temporal synchrony, indicating the presence of large-scale weather systems^8^. We also prioritized weather stations based on data completeness and higher monthly averages (Supplementary Figs. 2 and 3), as these better capture signals of extreme precipitation or anomalies^63^. Identified rainfall peaks were cross-validated through media reports and insights from local partners, and subsequently, used as input parameters to the FastFlood simulations. All data and model inputs required to reproduce the analysis, including rainfall analysis, DEM processing steps and model settings, are documented in the Supplementary Information. Input and output grid layers are openly available in the project repository (see “Data availability”).

Then, our flood modeling strategy started with a systematic sensitivity analysis in Nairobi, the pilot city. In this phase, over 15 model runs were conducted with pre-defined variations of key parameters. In the absence of high-quality validation data, we defined a set of criteria that guided the assessment of the model: (1) minimized visual noise, often associated with the DEM and channel settings^37^; (2) higher maximum flood depths for capturing damaging events^63^; (3) and flood extents spread beyond natural channels, thereby effectively representing pluvial flooding patterns^1^. The analysis demonstrated that increasing model complexity resulted in a significant escalation of computation time without evident improvements in output differentiation. It became harder to assess the differences between results, either because further parameter complexity led to equifinality or to model limitations to properly capture physical processes^64^. Based on this sensitivity analysis, a simplified modeling setup retained a set of core inputs and parameters across all cities. Specifically, we (i) excluded channel routing and depression-filling options that led to unrealistic inundation patterns, (ii) avoided high solver accuracy that substantially increased computational time without improving spatial differentiation, and (iii) prioritized settings that reproduced flooding patterns beyond main river channels. Tested parameters and extended calibration configuration are provided in Supplementary Tables 2 and 3. Together, these choices define a consistent flood hazard modeling workflow applied uniformly across case studies and ensuring reproducibility, particularly in SSA municipalities with limited technical resources.

For the final flood maps, we used three flood depth thresholds: 1 cm (due to the underestimation of FastFlood), 10 cm (natural break of the dataset and in accordance with international benchmarks^65^), 50 cm (where life-threatening impacts were reported by the residents of the deprived communities).

Flood hazard maps were generated using a 50 × 50 m grid. This grid was selected based on three main parameters from previous intra-urban studies in LMIC: the size of the input parameters, the computational time of the analysis and the depiction of settlements of organic (or non-geometrical) shapes^27–29^ (Supplementary Table 7). A grid cell is classified as exposed when the simulated flood depth exceeds a given threshold, and the cell contains built-up area from either GOB or GHSL.

Based on this grid, we estimated built-up density derived from two global datasets: the GOB layer (after computing building area sum per grid cell) and the GHSL layer. While other global layers offer higher resolution, like HRSL, GHSL was selected due to its global consistency, suitable for cross-city comparison^55^. At the same time, it has known limitations in capturing complex urban environments^31^. GOB, in contrast, provides an AI-derived footprint reported with one of the highest counts of buildings in African cities and improved sensitivity to small structures typical of DUAs in comparison to the Microsoft product^32,34^. A comparative analysis of these datasets to estimate flood exposure is, to our knowledge, yet to be implemented. As the built-up areas are the elements at risk of this analysis, we defined density thresholds to classify the built-up categories into low, medium and high categories based on the proportion of built-up area per grid cell. Thresholds were statistically derived from the data distribution and agreement between the GOB and GHSL, resulting in low (≤20%), medium (20–50%), and high (>50%) built-up density classes (Supplementary Figs. 4 and 5).

Flood exposure was then calculated by overlaying the flood hazard maps and the elements at risk datasets. We computed both absolute and relative exposure across cities and evaluated the results by flood depth category and built-up density class. Absolute exposure corresponds to the total built-up area (km^2^) intersecting flooded grid cells for a given flood depth threshold, and Relative exposure represents the percentage of built-up areas exposed to flooding, calculated as the ratio between exposed built-up area and total built-up within a city. These metrics were quantified for three flood depth thresholds, allowing comparison of shallow versus deeper flooding patterns. Lastly, we analyzed the differential exposure between deprived and non-deprived areas, also computing relative exposure within the cities.

**Fig. 8:**
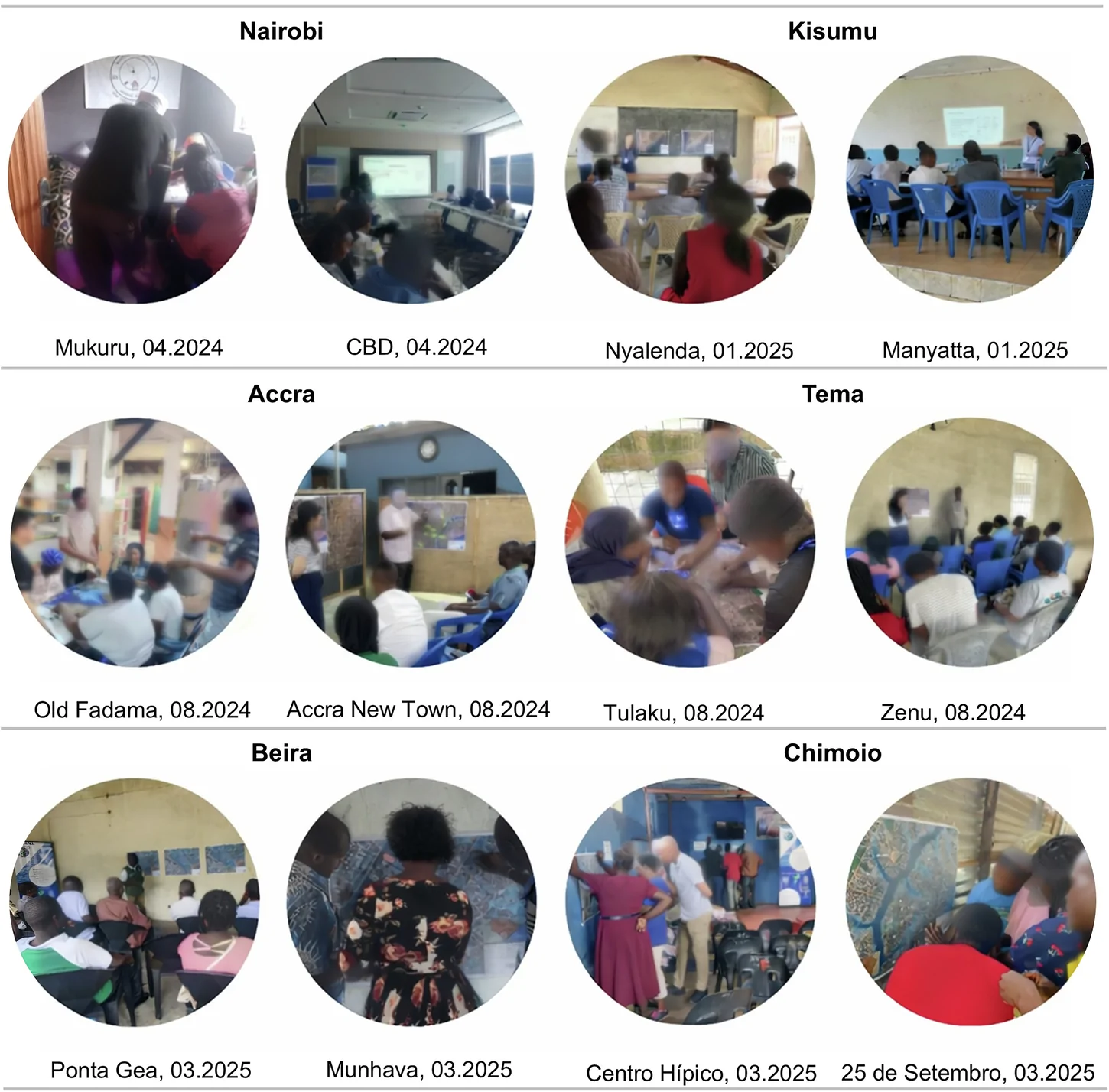
Workshops across the six study cities. The figure shows sample photographs from workshops conducted in Nairobi, Kisumu, Accra, Tema, Beira, and Chimoio. The panels show workshop settings of selected communities. Participants provided verbal and written informed consent (Supplementary Figs. 6 and 7). Identifiable faces have been blurred in accordance with ethical approved from the University GeoEthics Committee (application no. 240127).

Satellite-based products were explored as a validation source. In particular, flood extent maps produced by UNOSAT from PLEIADES imagery for the May 2024 flood event were examined for Nairobi. Visual comparison showed that the product primarily captured fluvial flooding in major rivers, failing to represent widespread pluvial flooding reported by DUA residents. Given the limitations of conventional validation data, such as these satellite products and observed discharge records, we integrated a citizen science-based approach, combining local workshops and participatory mapping surveys. In each city, we organized two sets of workshops: one at the city scale, involving city council representatives, NGOs and academia, and another at the community scale, engaging with residents of selected DUAs. Participants were recruited taking into account the diversity of age, gender and livelihood. To reduce power imbalance, group composition was managed to avoid dominance by a single leader or institutional actor. The number of visited communities was refined through fieldwork insights. We started with four communities in Nairobi, but subsequent iterations showed that two contrasting areas (in terms of morphology, stage of development, economic functions and location) provided sufficient insights. During the local workshops, the participants were divided into small groups (3–5 people)^66^ to assess printed flood maps by identifying flood-prone and safe spots and discrepancies between model outputs and the community experiences (Fig. 8). The group findings were then discussed in plenary, where they evaluated the level of agreement across the groups and consolidated insights. The citizen science protocols are further described in Supplementary Information.

At the community level, and refraining from the limited perspective of citizens as simply data collectors^67^, we engaged with leaders and residents from DUAs in Nairobi through a participatory process structured in three phases: (1) we codesigned a data collection protocol (Supplementary Fig. 8). Through a series of online focus groups, participants selected Google My Maps as the preferred tool (that can be used on mobile phones with limited mapping experience), and we co-created the survey content based on their information needs, ensuring the results were actionable for the communities^68^; (2) we trained the residents to map flood observation points (Fig. 8), where they documented flood intensity using locally defined categories: ankle (up to 15 cm), knee (up to 50 cm), waist (up to 100 cm) and chest (above 100 cm), along with descriptions of the impacts in each category; (3) we compared the collected observation points to the FastFlood outputs using hit-and-miss evaluation approach to assess the model performance. A “hit” was defined when the predicted class matched the observed class, or the observed class was only one ordinal level below the predicted class. We also spatially assessed the observation points to check sampling biases (Supplementary Fig. 9).

Additionally, because up-to-date spatial data on local drainage infrastructure is largely unavailable across the study areas, the flood model does not explicitly represent these systems. Instead, our understanding of infrastructure-related drivers is informed by the citizen science methodologies in fieldwork, which offered valuable perspectives on local processes such as drainage malfunction and informal modifications that are not captured in the model.

To better understand the reported impacts of shallow flooding (up to ankle height)—particularly where the model underestimated the flood intensity—we conducted a thematic analysis of the reported impacts from the 338 flood observation points (Supplementary Fig. 10). After excluding incomplete or ambiguous responses (Supplementary Table 9), 310 entries were coded. We derived 56 distinct impacts, which were grouped inductively and thematically into nine overarching impact themes based on the Sendai Framework (Table 3). To handle ambiguity and deal with more than one reported impact per point, the insights from the local workshops were used to guide the classification. Based on this thematic analysis, we had a solid basis to quantify the frequency of impact patterns across the flood depth categories.

**Table 3:** Citizen-reported flood impacts were grouped into nine standardized thematic categories

Impact themes	Grouped impacts
Access disruption	Road blockage, disruption of access, disruption of services/activities, closure of schools, damage to schools
Property damage	Home inundation, destruction of properties, property loss, damage to houses, destruction of houses
Infrastructure damage	Damage to structures, damage to roads, destruction of roads, destruction of facilities (playground, toilets), destruction of bridges
Environmental issues	Erosion, landslides, water pollution, environment pollution
Disease outbreak	Disease outbreak, water contamination, stagnant water, water-borne disease outbreak, malaria outbreak, mosquitoes, diarrhea
Urban farm losses	Crop damage, livestock loss, crop losses
Loss of life	Death, loss of lives
Displacement	Evacuation, displaced residents, “no residents”, destruction of property and displacement, “forced migration”, “people had to shift”
Destruction of property & loss of life	Destruction of houses and death, property destruction and loss of lives

The table presents the impact themes derived from thematic analysis of flood observations reported by residents of deprived urban areas through the participatory mapping survey. Columns list the overarching impact theme and the grouped impacts assigned to each theme.

## Supplementary information

Supplementary

## Data Availability

The custom Python scripts generated during the current study are available in the Zenodo repository named "Data and Code Repository - Flood Exposure Assessment in Sub-Saharan African cities", DOI 10.5281/zenodo.15648813. The input and output datasets that support the findings of this study are also made available in the same repository, under open data license.
