# Supplementary material for: Exposed yet unmapped: evidence of differential flood exposure in deprived urban areas using citizen science
Source: NPJ Urban Sustain. 2026 Jun 22;6(1):127. doi: 10.1038/s42949-026-00428-8 (PMC13649584; doi:10.1038/s42949-026-00428-8)
Supplement: Supplementary file 1 [file 42949_2026_428_MOESM1_ESM.pdf]

SUPPLEMENTARY INFORMATION

Exposed yet unmapped: Evidence of differential flood exposure in deprived urban areas using citizen science

Table of Contents

Flood proneness - intra-urban analysis 2
Flood inventory 2
Flood model experiments 4
Manual Workflows in QGIS 6
  Catchment Delineation 6
  SPI Computation 7
Rainfall Analysis in Excel 7
Statistical comparison of GOB and GHSL 9
Workshop Protocols 10
Participatory mapping 13
  Data collection and analysis 15
Content Analysis 17

Flood proneness - intra-urban analysis

This section compares the most flood-prone districts in each city with different flood depth thresholds. The results show that often the most flood-prone districts are exposed to all flood depths in Beira, Tema, Kisumu and Nairobi (Supplementary Figure 1). This finding indicates persistent flood patterns, where more exposed areas do not necessarily reduce by increasing flood depth.

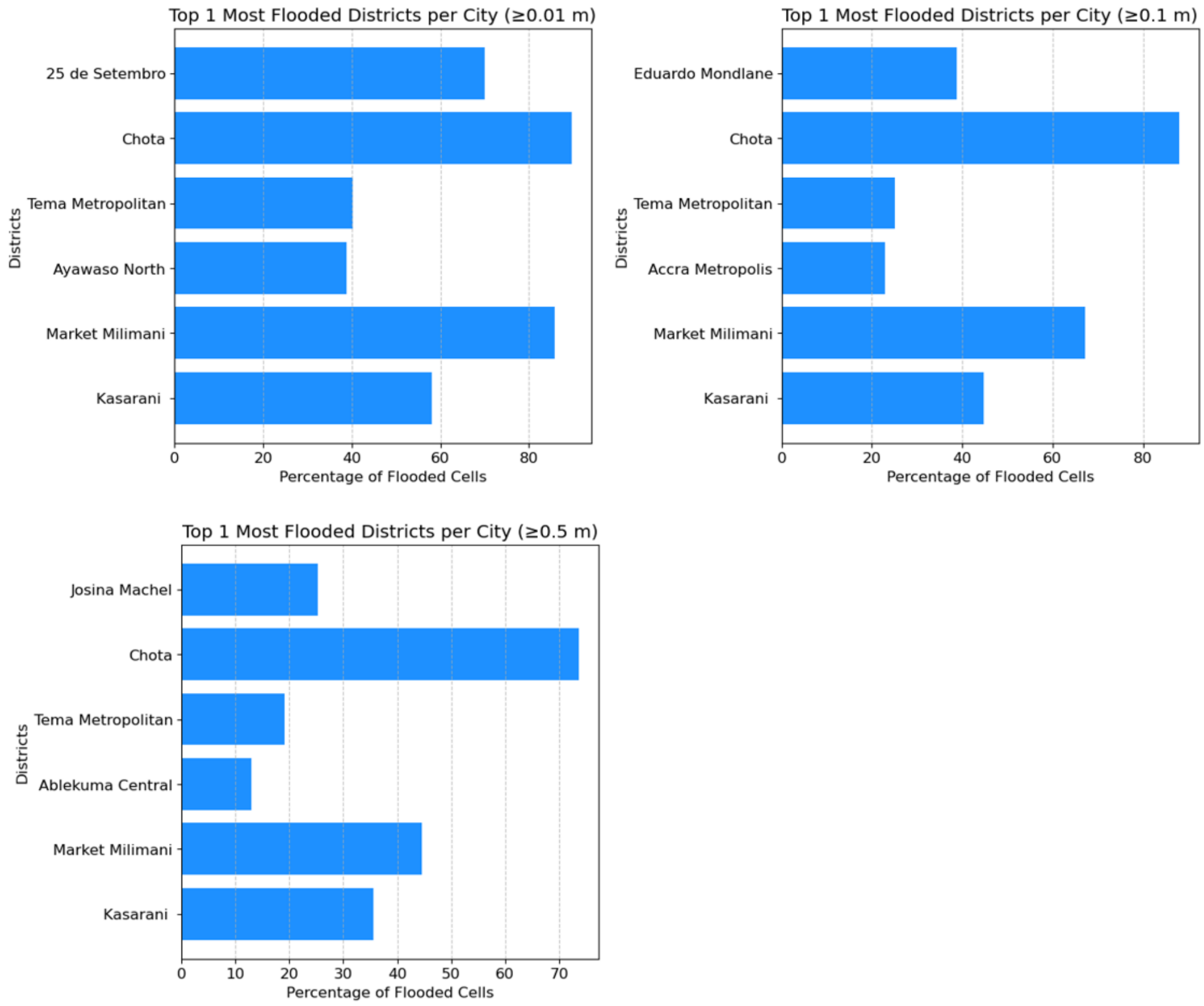

Supplementary Figure 1 - Most flooded districts per city in each flood depth threshold*. Horizontal bar charts show the five most flooded districts in each city at three modelled flood-depth thresholds: a) 0.05 m, b) 0.30 m, and c) 0.50 m. The x-axis shows the percentage of flooded cells within each district, and the y-axis lists the district names.*

Flood inventory

We had serious difficulties locating flood hazard maps and assessments at city scale for the study areas. When available in publications, responses for data acquisition were rare; when available in government reports, maps were provided at national scale and at city level only processed statistical data provided. At the same time, we could find multiple sources of aspatial flood information that highlighted the alarming historical patterns of flood in the cities, e.g., the Kenyan National Climate Change Action Plan report[1] and the Ghana's Fourth National Communication under the United Nations Framework Convention on Climate Change[2]. To understand the nature of flooding impacts in the study areas and select the events for flood modelling, we conducted a historical inspection for the last 20 years. Here verified flood events reported online for each city, through an extensive (non-exhaustive) search, and crosschecked with the Dartmouth Flood Observation (DFO) database and official records from relevant national authorities. We excluded online news articles that lacked information on impacts or did not explicitly mention the selected cities as affected areas. Supplementary Table 1 below is a summary of the findings. Although biased by the search in English, it is clear that evidence on secondary cities is not easily accessible, indicating less attention to these area at national scale, but also digital and technological disparities[3].

Location	Date	Impacts	Sources
Greater Accra	06/2023	8 fatalities	https://floodlist.com/africa/ghana-floods-june-2023
	03/2023	3 fatalities	Ghana – Accra Floods Cause Damage and Fatalities – FloodList
	06/2021	4 deaths, 6 injured, 200 displaced	https://erccportal.jrc.ec.europa.eu/ECHO-Products/Echo-Flash#/daily-flash-archive/4230
	04/2019	12 deaths	https://floodlist.com/africa/ghana-deadly-floods-accra-april-2019
	06/2018	5 deaths; over 500 displaced;	Ghana – Devastating Floods Hit Accra Again – FloodList
	06/2016	5 deaths	News (The Watchers) & FloodList Ghana – Deadly Floods Hit Central Region – FloodList
	06/2015	Explosion of Fuel Station caused by torrential rainfall (154 deaths)	https://www.theguardian.com/world/2015/jun/05/death-toll-accra-floods-petrol-station-fire
Beira & Chimoio	03/2024	over 11000 households displaced and 2 deaths	Mozambique - Maputo heavy rains and Tropical Storm Filipo - Flash Update No. 3 (28 March 2024) | OCHA
	03/2023	Over 200000 displaced, over 180 deaths	Mozambique: Floods - Feb 2023 | ReliefWeb
	01/2021	6 fatalities, 8000 displaced	Southern Africa – Tropical Cyclone Eloise Triggers Floods in Mozambique, Zimbabwe and South Africa – FloodList
	12/2020	Over 3 deaths	Mozambique – Flash Floods in Beira, Sofala Province, After Heavy Rain – FloodList
	03/2019	48 deaths (Mozambique)	Mozambique and Zimbabwe – Tropical Cyclone Idai Causes Death and Destruction – FloodList
Nairobi	04/2024	39 deaths, over 20000 displaced	At least 39 people killed, 20,000 displaced by floods in Nairobi<br>Kenya: Heavy Rains and Flooding Update - Flash Update #5 (10 May 2024) | OCHA
Kisumu	04/2023	4, 500 Households Displaced	https://www.kenyanews.go.ke/4-500-households-displaced-by-floods-in-kisumu/
Kisumu & Nairobi	04/2022	8,850 displaced households	https://reliefweb.int/report/kenya/kenya-floods-dref-operation-n-mdrke047-final-report
Kisumu & Nairobi	05/2021	Over 13000 families displaced; 9 fatalities	https://floodlist.com/africa/kenya-floods-update-may-2021
Kisumu	04/2020	13000 displaced	13,300 displaced in flood-hit parts of Kisumu County – Kenya News Agency
Kisumu	06/2018	Over 7000 people displaced	Kenya Floods Response Update – 8 June 2018, Flash Update - Kenya | ReliefWeb
Nairobi	04/2016	Over 10 deaths	https://floodlist.com/africa/kenya-floods-building-collapses-huruma-nairobi

Supplementary Table 1 – Historical flood inventory summarizing reported flood events in the study sites. *The table lists verified flood events identified through online reports, official records, and cross-checks with available flood databases. Columns report the affected location, event date, documented impacts, and source information.*

Flood model experiments

We split the FastFlood model experiments into two sets: 1) in the pilot city, experiments with different sets of input and calibration parameters(Supplementary Table 2); 2) the other five cities replicating the tests with core input datasets (Supplementary Table 3). We restricted the model simulation to core input after the simulations done in Nairobi for two main reasons. First, more complex model setup leads to an exponential escalation of computational time (and recurrent time out errors). Second, in the absence of validation reference sources, visual assessment is time-consuming and hard for interpretation. The hydrodynamic effects might be incomprehensible for city planners (targeted local stakeholders) and increasing usability of the modelling tool is important for this research. Considering a resource-prohibitive environment, with limited staff and computation capacity, we opt for a simplified calibration process.

Input Data	Calibration Parameters	Output Remarks
SRTM 40m		Computational Time (CT) = under 30s<br>Max Flood Depth (MFD) = 19m
SRTM 40m<br>WorldCover 10m		CT = under 30 seconds<br>MFD = 22m
SRTM 40m<br>WorldCover 10m<br>Soilgrids (5-15cm)		CT = under 30s<br>MFD = 22m
SRTM 40m<br>WorldCover 10m<br>Soilgrids (5-15cm)<br>Channels (Automatic)		CT = under 30s<br>MFD = 40 m
Copernicus 40m<br>WorldCover 10m<br>Soilgrids (5-15cm)		CT = under 30s<br>MFD = 30m
Copernicus 40m<br>WorldCover 10m<br>Soilgrids (5-15cm)<br>Channels (Automatic)		CT = under 30 seconds<br>MFD = 46m<br>Visualization (VIZ) = less pixalized, better depiction of channels
Copernicus 40m<br>WorldCover 10m<br>Soilgrids (5-15cm)<br>Rainfall (Nov/2019)		CT = under 30 seconds<br>MFD = 22m
Copernicus 40m<br>WorldCover 10m<br>Soilgrids (5-15cm)<br>Rainfall (Nov/2019)	Flat terrain activated	CT = under 90s<br>MFD = 29m<br>VIZ = higher depth and spread streams and water bodies
Copernicus 40m<br>WorldCover 10m<br>Soilgrids (5-15cm)<br>Rainfall (Nov/2019)	Flat terrain activated<br>Output discharge<br>Account for depressions	CT = under 120s<br>VIZ = pixelated results
Copernicus 40m<br>WorldCover 10m<br>Soilgrids (5-15cm)<br>Channel (Automatic)<br>Rainfall (Nov/2019)	Flat terrain activated	CT = under 120s<br>MFD = 37m<br>VIZ= whole city exposed (unrealistic)
Copernicus 40m<br>WorldCover 10m<br>Soilgrids (5-15cm)<br>Channel (Automatic)<br>Rainfall (Nov/2019)	Flat terrain activated<br>Output discharge<br>Account for depressions	CT = under 150s<br>MFD = 32m<br>VIZ = unflooded depression patches

Copernicu 40m<br>WorldCover 10m<br>Soilgrids (5-15cm)<br>Channel (Multipliers)<br>Rainfall (Nov/2019)	Flat terrain activated	CT = 160 seconds<br>MFD = 27m<br>Channels are not part of a flood mitigation network (unrealistic)
Copernicus 40m<br>WorldCover 10m<br>Soilgrids (5-15cm)<br>Channel (Multipliers)<br>Rainfall (Nov/2019)	Flat terrain activated<br>Solver Accuracy (High)	CT = 7 minutes<br>MFD= 33m<br>VIZ = larger flood extent and depth compared to model 14

Supplementary Table 2 - FastFlood experiments conducted in Nairobi*. The table summarizes FastFlood model experiments conducted for the pilot city. The first column lists input datasets, the second lists calibration parameters and the third summarizes output remarks. The blue-highlithed row identified the selected model setting.*

City	Input Data	Output Remarks
Kisumu	Copernicus GLO 40m<br>WorldCover 10m	
	SRTM 40m<br>WorldCover 10m	VIZ = Salt and pepper effects<br>Lower MFD
	Copernicus GLO 40m<br>WorldCover 10m<br>Soilgrids (5-15cm)	VIZ = Flood extent spread outside major rivers (more realistic)
	Copernicus GLO 40m<br>WorldCover 10m<br>Soilgrids (5-15cm)	MFD = 10m<br>VIZ = Larger flood extent (into minor waterways too)
	Copernicus GLO 40m<br>WorldCover 10m<br>Soilgrids (5-15cm)<br>Rainfall (May/2021)	Inclusion of rainfall cut MFD in half max flood depth reduced to half (5.5m)
	Copernicus GLO 40m<br>WorldCover 10m<br>Soilgrids (5-15cm)<br>Rainfall (May/2021)<br>Flat terrain	MFD = 8.5m<br>VIZ = whole city exposed (unrealistic)
	Copernicus GLO 40m<br>WorldCover 10m<br>Soilgrids (5-15cm)<br>Rainfall (May/2021)<br>Channel (Automatic)<br>Flat terrain	MFD = 9.5m<br>VIZ = DEM resolution issues lead to unrealistic simulated channels
Accra & Tema	Copernicus GLO 40m<br>WorldCover 10m	
	SRTM 40m<br>WorldCover 10m	VIZ = salt and pepper and reduced flood extent
	Copernicus GLO 40m<br>WorldCover 10m<br>Soilgrids (5-15cm)	VIZ = Larger flood extent overall also into minor waterways
	Copernicus GLO 40m<br>WorldCover 10m<br>Soilgrids (5-15cm)<br>Rainfall (Apr/2022)	Minimal change, slightly larger MFD
	Copernicus GLO 40m<br>WorldCover 10m<br>Soilgrids (5-15cm)<br>Rainfall (Apr/2022)<br>Flat terrain	VIZ = whole city exposed (unrealistic)
Beira	Copernicus GLO 40m	MFD = 6.3m
	SRTM 40m	VIZ = salt and pepper<br>Lower MFD

	Copernicus GLO 40m<br>WorldCover 10m	MFD = 4.9m<br>VIZ = Reduced flood extent
	Copernicus GLO 40m<br>WorldCover 10m<br>Soilgrids (5-15cm)	VIZ = Flood extent spread outside major rivers (more realistic)
	Copernicus GLO 40m<br>WorldCover 10m<br>Soilgrids (5-15cm)<br>Rainfall (Apr/2019)	VIZ = Larger flood extent overall also into minor waterways
	Copernicus GLO 40m<br>WorldCover 10m<br>Soilgrids (5-15cm)<br>Rainfall (Feb/2021)	VIZ = less extent and more depth close to main riverways (less realistic)
	Copernicus GLO 40m<br>WorldCover 10m<br>Soilgrids (5-15cm)<br>Rainfall (Apr/2019)<br>Flat Terrain	VIZ = whole city exposed (unrealistic)
Chimoio	Copernicus GLO 40m	MFD = 24 m
	SRTM 40m	MFD = 20m<br>VIZ = not so different from GLO
	Copernicus GLO 40m<br>WorldCover 10m	MFD = 22 m<br>VIZ = Reduced flood extent
	Copernicus GLO 40m<br>WorldCover 10m<br>Soilgrids (5-15cm)	VIZ = Flood extent slightly in main riverways
	Copernicus GLO 40m<br>WorldCover 10m<br>Soilgrids (5-15cm)<br>Rainfall (Apr/2019)	MFD = 26.5m<br>VIZ = larger flood depth and extent
	Copernicus GLO 40m<br>WorldCover 10m<br>Soilgrids (5-15cm)<br>Rainfall (Apr/2019)<br>Flat terrain	MFD = 21m<br>VIZ = western part has increased flood extent, raising pressure into Revue catchment and affecting the city (realistic)

Supplementary Table 3 - FastFlood Experiments in the other five cities. *The table summarizes FastFlood experiments for Kisumu, Accra and Tema, Beira, and Chimoio. For each city, the table lists tested input datasets and output remarks. Blue-highlighted rows indicate the model configurations retained.*

Manual Workflows in QGIS

This section documents the step-by-step procedure used to delineate the city-cale catchment boundaries for each study area and to compute the Strem Power Index that guided the selection of deprived urban settlements. All operations were executed in QGIS 3.14 “Pi” on Windows 10.

Catchment Delineation

Before the collection of input layers (Supplementary Table 4), we conducted a desk review, collating catchment names and extent from government reports and peer-reviewed articles. Then, we downloaded the catchment layer from HydroBaSINS and GADM for each city and visually overlaid them, inspecting which basin level enclosed the entire city with minimal external upstream area. Then, we retained the smallest level that fully covered the GADM boundary still ensuring upstream contributions. For reassurance and crosscheck at city level, we downloaded the SRTM DEM and employed a workflow using SAGA GIS tools to derive our own catchments. We followed the steps presented in Supplementary Table 5. The final extent used for the FastFlood modelling are included in the shared Zenodo repository (10.5281/zenodo.15648813).

Layer	Properties	Source
HydroBASINS levelled polygons	Levels 3–10	https://www.hydrosheds.org/products/hydrobasins
GADM administrative limits		https://gadm.org/
SRTM DEM	2014	QGIS plug-in "SRTM-Downloader"
Google Satellite	—	QGIS plug-in "Quick Map Services"

Supplementary Table 4 - Input data for catchment delineation, using HydroBASINS, administrative boundaries, elevation data, and satellite imagery in QGIS. *Columns report the layer name, relevant properties, and data source.*

Step	QGIS / SAGA tool	Key parameters
1	Fill sinks (Wang & Liu)	Minimum slope = 0.01 %; Method = Wang & Liu
2	Generate hillshade	Azimuth = 315°, Altitude = 45°
3	Flow accumulation	Method = Deterministic 8
4	Channel network & drainage basins	Strahler order threshold (city dependent. The threshold chosen after visual comparison with Google Satellite Imagery.

Supplementary Table 5 – QGIS tools used to derive catchment boundaries from SRTM elevation data using QGIS. *The table summarizes the processing steps used to generate catchment extents from Shuttle Radar Topography Mission digital elevation model data (SRTM DEM).*

SPI Computation

The Strem Power Index was calculated using the SRTM DEM and following the four steps below (Supplementary Table 6). The flow accumulation raster (flow_accum_cell) was produced in cell units and the slope raster (slope_deg) was converted to radians inside the raster calculator expression for consistency with the SPI formula. The output SPI raster is a floating tiff where higher values indicate locations with both large upstream contributing areas and steep gradients- interpreted as prone to greater erosive power and floods. The rasters were intersected with polygons of deprived urban areas to identify zones with high flow accumulation and steep slope. These spots confirmed by local partners guided the selection of communities to join the citizen science methods.

Step	QGIS / SAGA tool	Key parameters / notes
Fill sinks	*SAGA → Terrain Analysis – Pre-processing → Fill Sinks (Wang & Liu)*	Minimum slope = 0.01 %
Flow accumulation	*SAGA → Terrain Analysis – Hydrology → Flow Accumulation (Top-Down)*	Method = Deterministic 8; Output unit = Cells
Slope (degrees)	*SAGA → Terrain Analysis – Morphometry → Slope, Aspect, Curvature*	Slope unit = Degrees
Stream Power Index	*QGIS Raster Calculator*	Expression: ln(("flow_accum_cell" + 1.0) * tan(radians("slope_deg")))

Supplementary Table 6 - Stream Power Index computation. *The table lists the SAGA GIS and QGIS tools used to compute the Stream Power Index (SPI). Columns report the processing step, tool name, and key parameters or notes.*

Rainfall Analysis in Excel

To process and analyze the hourly rainfall observations from TAHMO gauge stations, we performed a series of procedures in Microsoft Excel. The input data from TAHMO contained folders from each available station wich CSV files containing a timestamp and the precipitation in mm. The format of the time stamp was DateTime combining date and time (MM-DD-YYYY

HH:MM). We organized the data by a master workbook named Rainfall_Analysis with one sheet per station for each city. To aggregate the hourly measurements to daily totals we:

1) Create a new column Date with the temporal coverage of the data (1-1-2019 to 1-1-2024).
2) Create a new column “Daily Precipitation” and calculate the daily totals sing SUMIF function
3) Inspect (using Filter function) the top measurements and create plot chart for visual inspection of the rainfall hourly and daily peaks for each available station (see Supplementary Figure 2 as example)
4) Create bar chart of visual inspection of daily rainfall throughout the yearly temporal coverage (Supplementary Figure 3)
5) Check completeness of the stations by counting missing hourly records using COUNTBLANK function
6) Check synchrony of the peak dates across available stations using COUNTIF formula

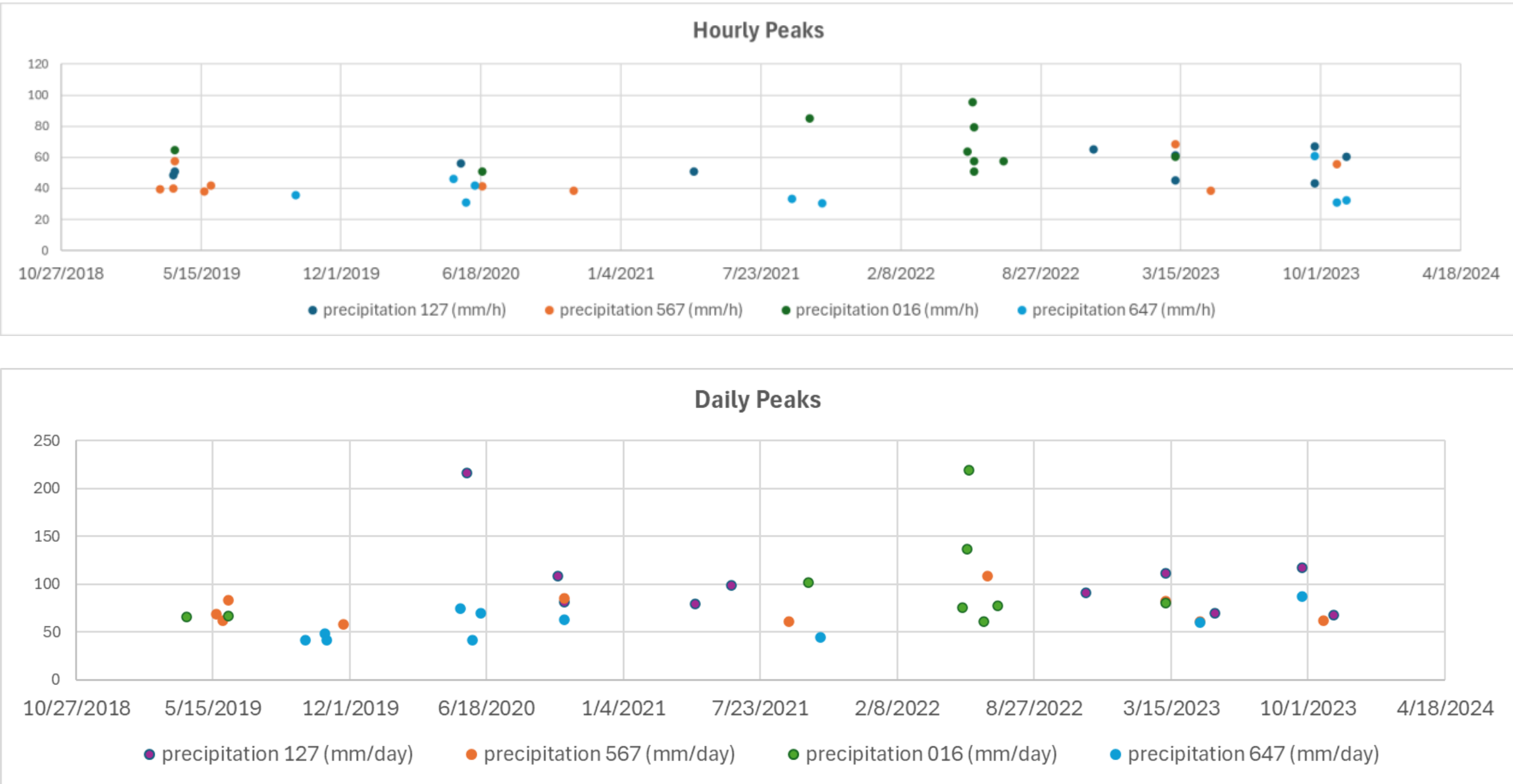

Supplementary Figure 2 - Hourly and daily rainfall peaks were identified from four TAHMO gauge stations around the Accra Metropolitan Area. *P The upper panel shows hourly precipitation peaks, and the lower panel shows daily precipitation peaks. The x-axis shows the date, and the y-axis shows precipitation in millimeters. Colored points distinguish between the individual TAHMO gauge stations, as indicated in the figure legend.*

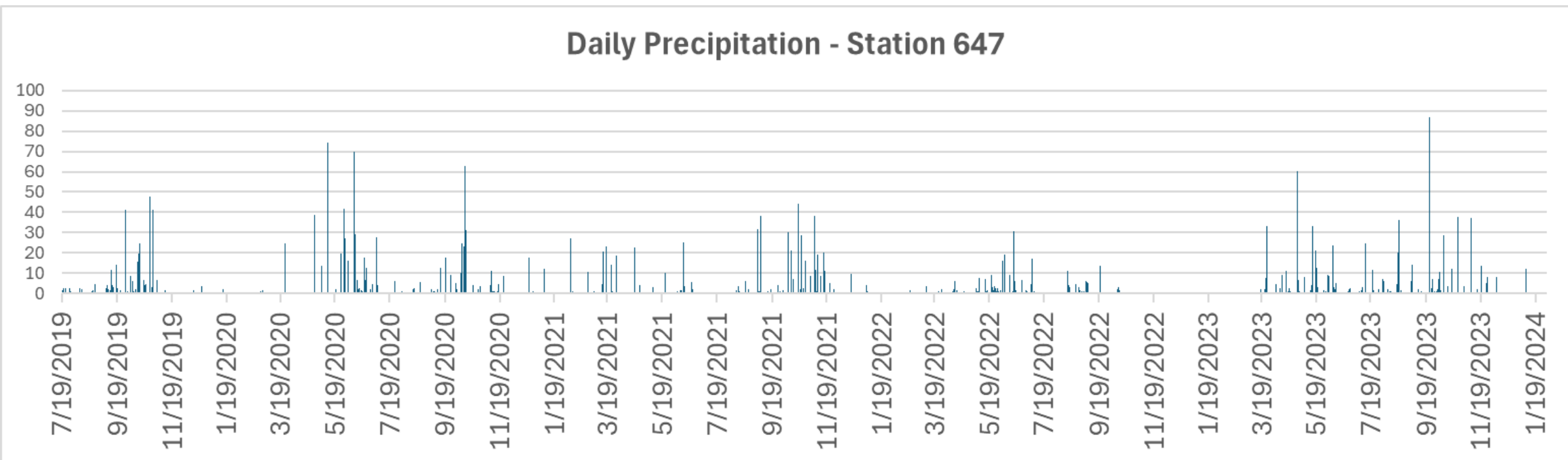

Supplementary Figure 3 - Daily precipitation records reveal rainfall variability. *The vertical bar chart shows daily precipitation totals for TAHMO Station 647 in Ghana across the available observation period. The x-axis shows dates, and the y-axis shows daily precipitation in millimetres. Light-blue vertical bars represent daily rainfall totals, with taller bars indicating higher precipitation.*

For cross-validation, media reports of flood incidents and local partners were consulted to match the peak dates found in the analysis for each city. The input data of the chosen stations are shared in the online Zenodo repository (10.5281/zenodo.15648813), each sheet per city. We thank the Trans-African Hydro-Meteorological Observatory (TAHMO) for the provision of meteorological data. Interested parties may contact info@tahmo.org.

Statistical comparison of GOB and GHSL

Built-up density approach – the higher the built-up density, the higher the exposure. Three categories (low, medium and high) were statistically derived from the level of agreement between the two datasets and their data distribution patterns (Supplementary Table 7). From Supplementary Figure 4, we noticed a high dominance of non- to low-built up areas in both datasets from the right-skewed distribution. At first sight, around 500m2 (20% of the cell is built-up) would be an adequate threshold for low density category and 1000m2 (40%) for high density. We tested a range of thresholds following this data pattern globally and individually – per city. In Supplementary Figure 5, we got the highest level of agreement between the datasets through the following thresholds: Low (up to 20% built up), Medium (up to 50% built up), High (up to 100% built up).

Dataset	Count	Mean	Std Dev	Min	25%	50%	75%	Max
GOB	32668	268	256	~0	70	205	394	2500
GHSL	41558	427	397	~0	44	305	790	2016

Supplementary Table 7 - Descriptive statistics of GOB and GHSL built-up area estimates. *The table reports summary statistics for built-up cells derived from Google Open Buildings (GOB) and the Global Human Settlement Layer (GHSL). Columns include count, mean, standard deviation, minimum, 25th percentile, median, 75th percentile, and maximum built-up area values. Values are reported for built-up cells only.*

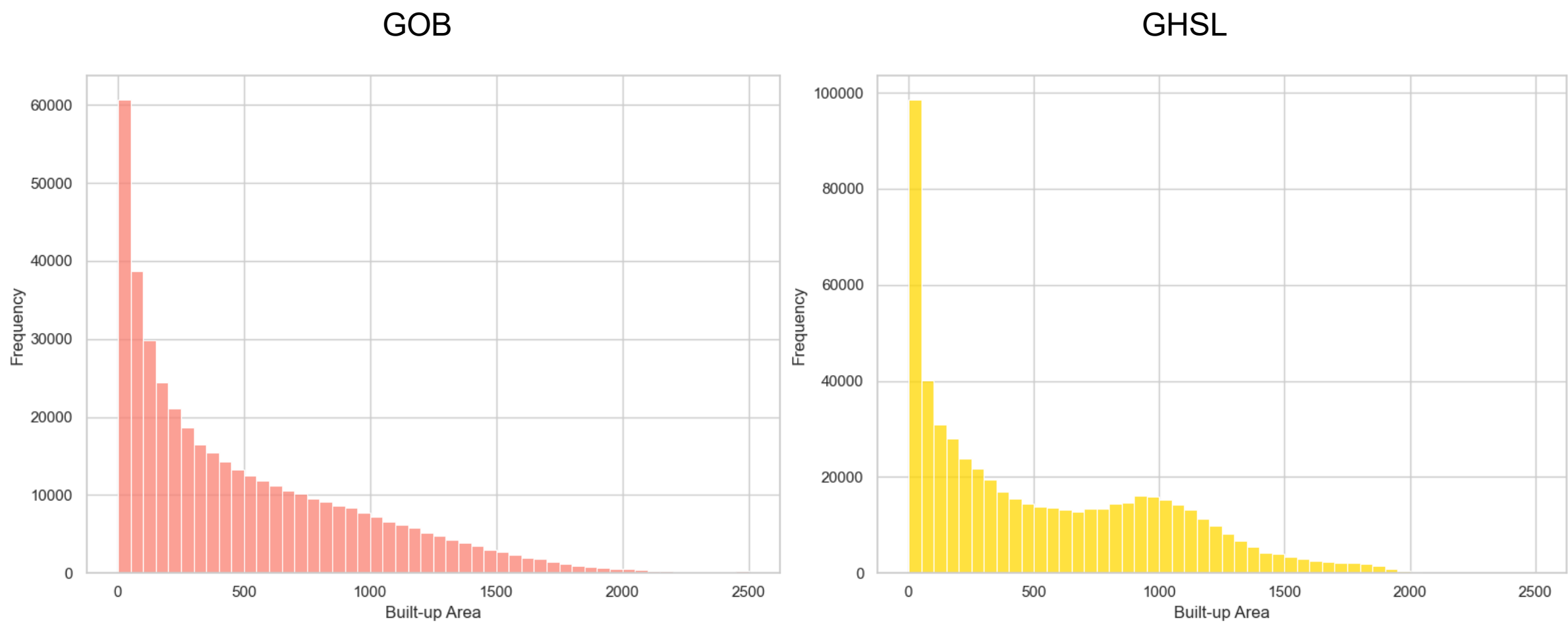

Supplementary Figure 4 - Built-up area estimates from GOB and GHSL. *The x-axis shows built-up area per 50 m by 50 m grid cell, and the y-axis shows frequency. Pink bars represent the GOB distribution, and yellow bars represent the GHSL distribution. Non-built-up cells were excluded from both distributions.*

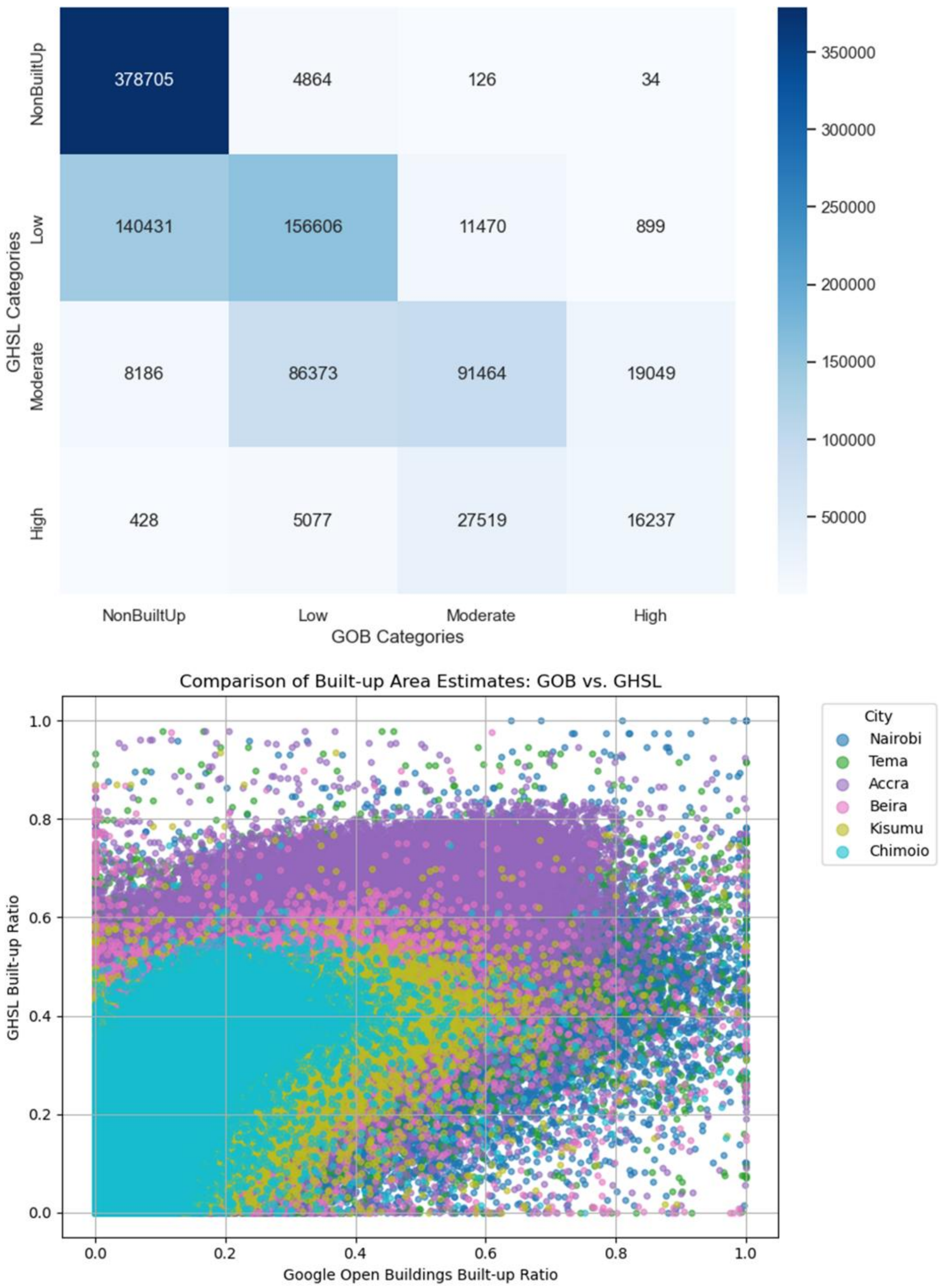

Supplementary Figure 5 - GOB and GHSL built-up density classes show 70% agreement. *The upper panel shows a confusion matrix comparing built-up density categories derived from GHSL (rows) and GOB (columns). Categories include non-built-up, low, moderate, and high built-up density. Darker blue cells indicate a larger number of grid cells in each category combination, while lighter blue cells indicate fewer grid cells. The lower panel shows a scatterplot comparing GOB built-up ratio on the x-axis and GHSL built-up ratio on the y-axis for each city. Colored points represent individual grid cells, with colors distinguishing the cities.*

Workshop Protocols

This part documents the workshop protocol used during fieldwork, including materials, facilitation techniques, programme structure and informed consent forms. Two workshop formats were conducted: (1) city-scale workshops, engaging with institutional actors (city planners, NGOs, local academia), and (2) community-scale workshops, involving residents of

deprived urban settlements. While the same protocol was followed in both formats, logistical arrangement differed. City-scale workshops were typically help in formal venues such as hotel lounges or city hall and did not include financial compensation for participants aside from lunch and transport. In contrast, community workshops were hosted in a variety of accessible local spaces-including churches, schools and even boxing clubs- to accommodate participants availability and comfort. Recognizing the economic issues of many participants, these workshops included financial compensation in addition to lunch and transport.

As part of a broader project, each workshop agenda was divided into three main sections (Supplementary Table 8). The sequence was adjusted based on the time of arrival of most participants. Regardless of the order, all sessions began with a warm welcome and verbal presentation of the printed informed consent form (see Supplementary Figure 6). Translation was provided by local partner or team member fluent in the native language.

FLOOD MODEL ASSESSMENT

**Time**	**Activity**
09h00-9h30	Welcome + Consent forms + Project Intro
9h30-10h	Tutorial
10h00-11h	Validation exercise
11h-12h	Plenary Discussion
12h-13h	Lunch break

LIVABILITY VOTING

**Time**	**Activity**
13h-13h15	Introduction
13h30-14h30	Voting app
14h30-15h	Discussion
15h-15h15	Break

VULNERABILITY IMPACT CHAINS

**Time**	**Activity**
15h15-15h45	Introduction
15h45-16h30	Impact chains co-design
16h30-17h	Feedback and closing remarks

Supplementary Table 8 - Programme of the workshops. *The table shows the programme structure used during the workshops. The agenda is divided into three color-coded sections: the yellow section shows Flood Model Assessment activities, the orange section shows Livability Voting activities, and the pink section shows Vulnerability Impact Chain activities. Grey rows indicate breaks or lunch periods.*

In the Flood Model Assessment section (the one pertaining to this paper), the participants were split into small groups of up to five people. Groups were composed to ensure diversity of gender and age, while also accounting for power dynamics (only one community leader or government representative of the same department in each group). One group in each session worked independently (without facilitator guidance) to minimize our influence and reveal unprompted insights. A brief tutorial (30 minutes) introduced participants to basic map-reading skills and cartographic features of the printed flood maps. Three orientation questions were asked in sequence: Where is the city hall? Where are we right now? Where is your house?. Then, participants were given approximately 120 minutes for the main activities.

- Map marking and impact annotations: using pins and marker pens, groups marked flood-prone and safe locations on the printed flood maps. Using sticky notes, groups described types of floods or past impacts experienced in the locations they mapped. In Supplementary Figure 7, we show example of such outputs.
- Plenary discussion: each group presented what they marked and shared insights with others. This process allowed us to cross-check agreement between groups (internal triangulation).

UNIVERSITY OF TWENTE.

INFORMATION LETTER/ INFORMED CONSENT FORM

Researcher(s)[redacted]

Introduction: Slums and informal areas face major challenges in [redacted] including inadequate housing and sanitation, while suffering severe consequences of flood events. Yet, there is little evidence and information on them.

This workshop has three main objectives:

(1) to understand your view of what a more livable area is. After familiarizing yourselves with satellite imagery and with a tool, you will be shown two images from different areas and will choose which one they would rather live in.

(2) to map and assess where flood events happen. Collectively, you will evaluate the results of the flood map we´ve simulated, discussing the wrong and the correct flood spots.

(3) To identify direct and indirect flood impacts in your neighborhoods

This research is community-led, we will understand floods and livability through your lenses. Your knowledge and experience will guide the data collection and outcomes. We hope the information generated today can benefit you.

Procedures: If you agree to participate, you will be asked to:

1) Share your preferences on better places to live.
2) Share your flooding experiences and observations about past and recent events.
3) Share your experience in flood direct and indirect impacts.

You are not required to share information that you are not willing to share.

All the information provided during this study will be treated as confidential. As this is for academic purposes, this data will only be accessed by us.

Your participation in this research is voluntary, you will not be forced to participate, and you have the right to opt out at any time.

If you have any questions, please feel very free to ask us.

By signing, you acknowledge that you have read and understood the information provided in this form, voluntarily agree to participate in the research study and consent the use of photos taken during the workshop for dissemination purposes.

UNIVERSITY OF TWENTE.

	Name	Signature	Cell Phone No.
1			
2			
3			
4			
5			
6			
7			
8			
9			
10			
11			
12			
13			
14			
15			
16			
17			
18			

Supplementary Figure 6 - Consent form template. *The figure shows the informed consent materials used during workshop activities. The left panel presents the participant information letter and consent form text, including the study purpose, voluntary participation statement, confidentiality information, and contact details. The right panel shows the signature sheet.*

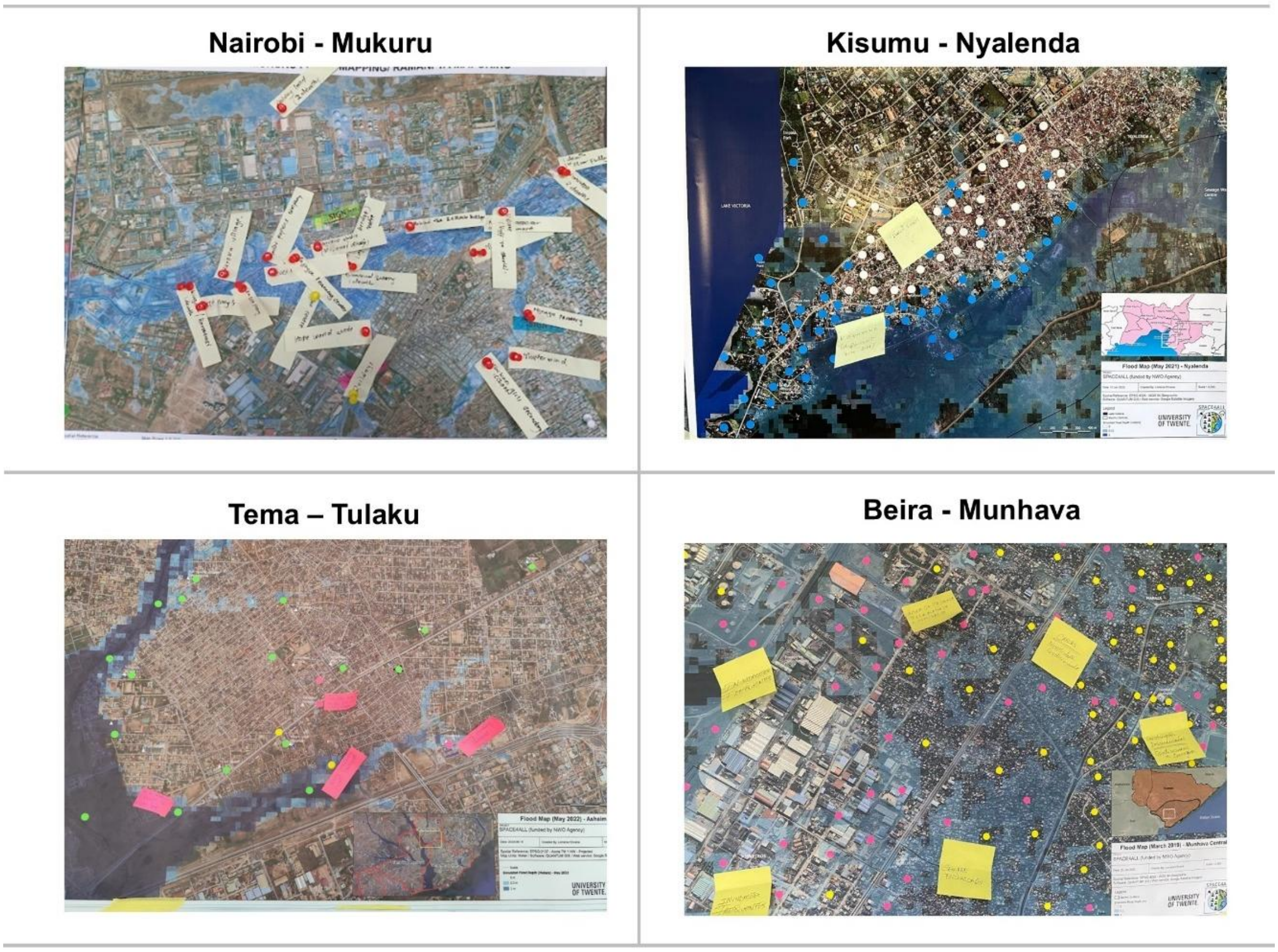

Supplementary Figure 7 - Community workshop outputs. *The four panels show examples of annotated printed flood maps produced during Flood Model Assessment sessions in community workshops: a) Nairobi–Mukuru, b) Kisumu–Nyalenda, c) Tema–Tulaku, and d) Beira–Munhava. Satellite imagery is used as the basemap in each panel. Colored pins, marker annotations, and sticky notes represent participant markings of flood-prone locations, safe areas, flood pathways, and locally reported impacts.*

Participatory mapping

Flood observation data were collected through a participatory citizen science approach co-developed within the Space4All project in collaboration with CommunityMappers (local organization of residents from Kibera linked to SDI Kenya). The methodology was designed to be participatory. To quantitively validate the flood model, we employed a community-based data collection approach, co-created in close collaboration with residents of Kibera community in Nairobi, which served as the pilot city of the project. This co-creation process ensured that the survey questions were relevant to the local context and addressed information needs of deprived communities. Key considerations during this phase included the practicality of the tool, clarity and comprehensibility of the questions. The data collection protocol was implemented using the Google MyMaps platform, allowing participants to geolocate and describe flood events in their communities. A snapshot of the My Maps survey interface used in Nairobi is provided in Supplementary Figure 8. To ensure applicability, the survey was translated to Portuguese for use in Mozambican cities.

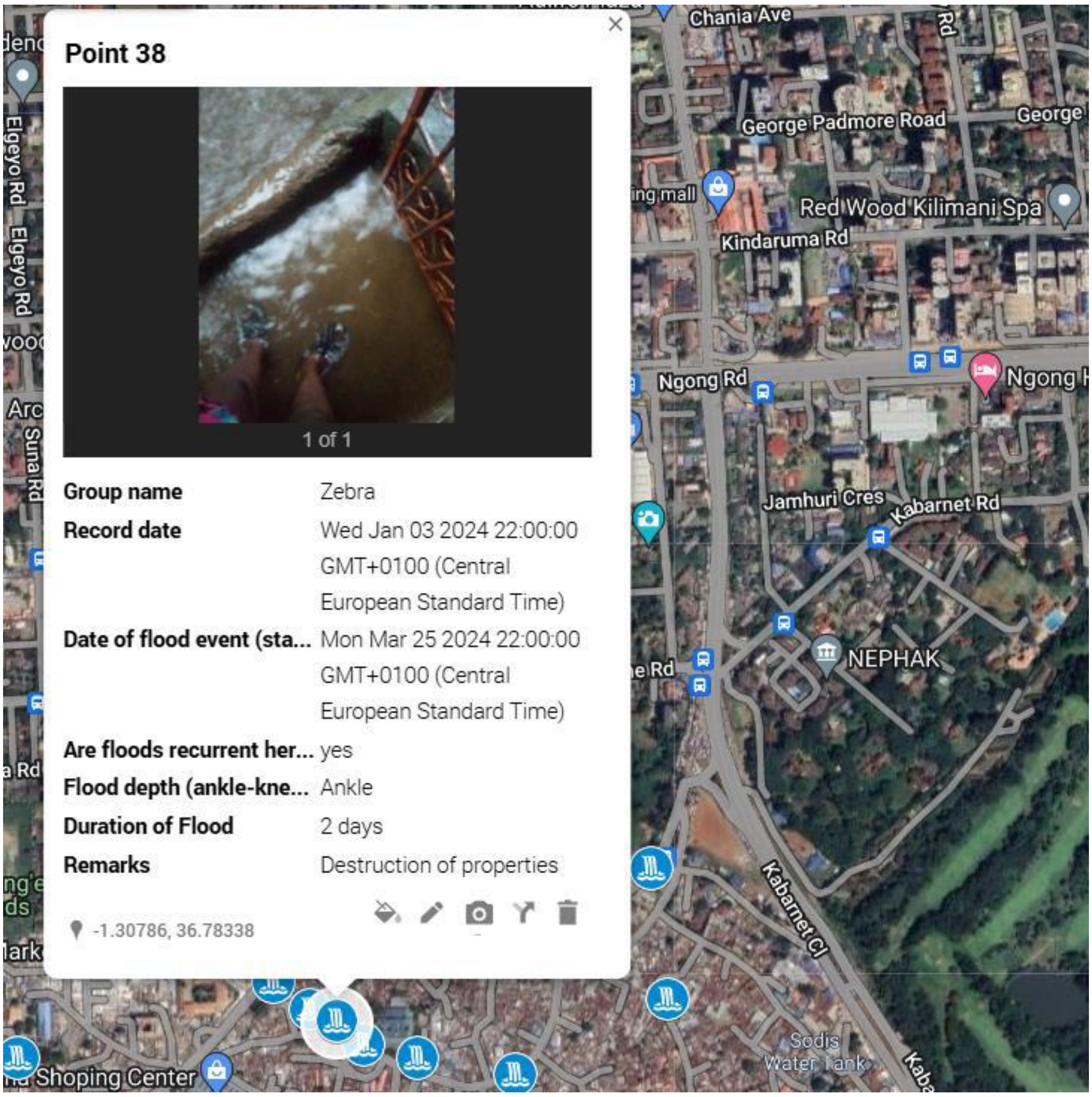

Supplementary Figure 8 – Screenshot of the Google My Maps survey interface used to collect geolocated flood observations*. The satellite basemap displays the mapped area, while the point pop-up window shows an example flood observation record.*

Following initial user information meetings in Nairobi (April 2024), two online testing and validation sessions were conducted to refine the data collection process and ensure usability. Considering local constraints such as limited access to technology and energy, in-person training sessions were carried out with selected representatives (two participants per community), who then supported broader community engagement.

Training combined digital and analog methods. Printed maps were used in a recognition exercise to familiarize participants with spatial features and locations prior to using the digital platform. Participants were then introduced to Google My Maps through a guided tutorial adapted from Data4HumanRights materials[4]. During these sessions, participants co-developed the survey structure, which included information on flood event timing, perceived flood depth, and associated impacts.

Participants were subsequently trained to conduct field data collection, including how to geolocate observations, classify flood depth using locally defined categories (ankle, knee, waist, chest), and document qualitative impacts. An online messaging group was established to provide ongoing support, address technical issues, and facilitate feedback throughout the data collection period. The survey was conducted by participants working in pairs, using their own mobile devices, fostering discussion and mutual support during fieldwork. The duration of the survey ranged from one to three hours, influenced by factors such as the extent they

covered and the energy of the pair. During the training, the pairs reflected on past flood events and planned the route to be conducted.

Participants involved in the mapping activities were selected during the workshop sessions in each community. Selection was guided by a combination of practical and participatory criteria. Given the digital nature of the data collection, preference was given to individuals who owned functioning Android smartphones and demonstrated basic technological familiarity, ensuring they could effectively use the Google My Maps platform. As a result, participants were predominantly younger adults, typically under 40 years old. Local community leaders were consulted to suggest potential participants; however, final selection also considered voluntary interest and availability, with participants invited on-site following the workshops. Efforts were made to achieve gender balance, with a target of equal representation. In practice, participation was slightly skewed towards men (approximately 60/40), particularly in secondary cities, reflecting local participation dynamics.

Data collection and analysis

Flood observations were collected and stored as point data within Google My Maps and later exported for integration with model outputs. Observed flood depths were standardized into locally defined categories and converted into metric values for analysis: ankle (up to 15cm), knee (up to 50cm), waist (up to 100cm) and chest (above 100cm).

For data quality, the submitted observations were reviewed for completeness and internal consistency in real time. During the initial data collection phase in Nairobi, we identified bias in the reporting of event dates, as this information relied on individual recall of when and how long flood events occurred. Given the associated uncertainty, these were not included in the analysis. The number of observations collected per city and community are reported in the Zenodo repository (DOI 10.5281/zenodo.15648813).

In addition, the spatial distribution of observation points was assessed to identify potential sampling biases. A consistent pattern across cities was the underrepresentation of points within river channels and immediate riverbanks, as participants typically mapped observations along accessible and walkable areas. Furthermore, local insights indicated that drainage pathways and streamlines are frequently altered by informal construction, which may not be accurately captured by the DEM. These factors were considered during interpretation of results, especially in comparison to model outputs. Supplementary Figure 9 illustrates the distribution of flood observation points across cities to exemplify possible spatial biases.

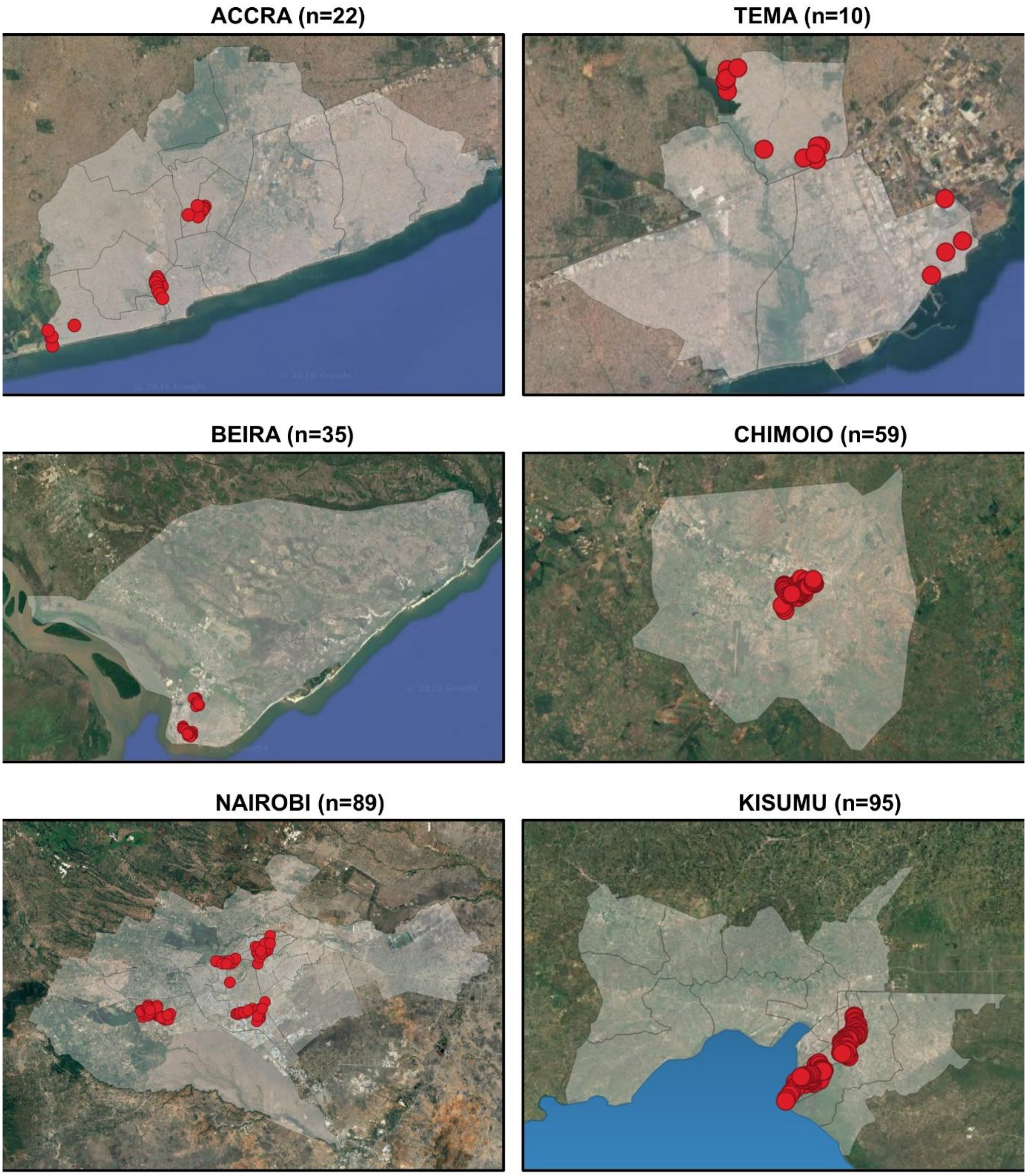

Supplementary Figure 9 - Spatial distribution of flood observation points across cities. *The six map panels show the spatial distribution of citizen-science flood observation points in a) Accra, b) Tema, c) Beira, d) Chimoio, e) Nairobi, and f) Kisumu. Red circles represent individual flood observation points collected by community participants. Panel titles report the city name and the number of collected points, where n indicates the point count.*

The flood observation points were also quantitatively assessed, compared with modelled flood depth using hit-and-miss evaluation approach adapted for ordinal flood intensity classes. Model outputs were first categorized into corresponding depth classes, and a “hit” was defined when the predicted class matched the observed class or observed class is only one ordinal level below the predicted class (Supplementary Figure 10). This tolerance accounts for uncertainty in both modelled depths and perception-based reporting of flood intensity. All other cases were considered a “miss”.

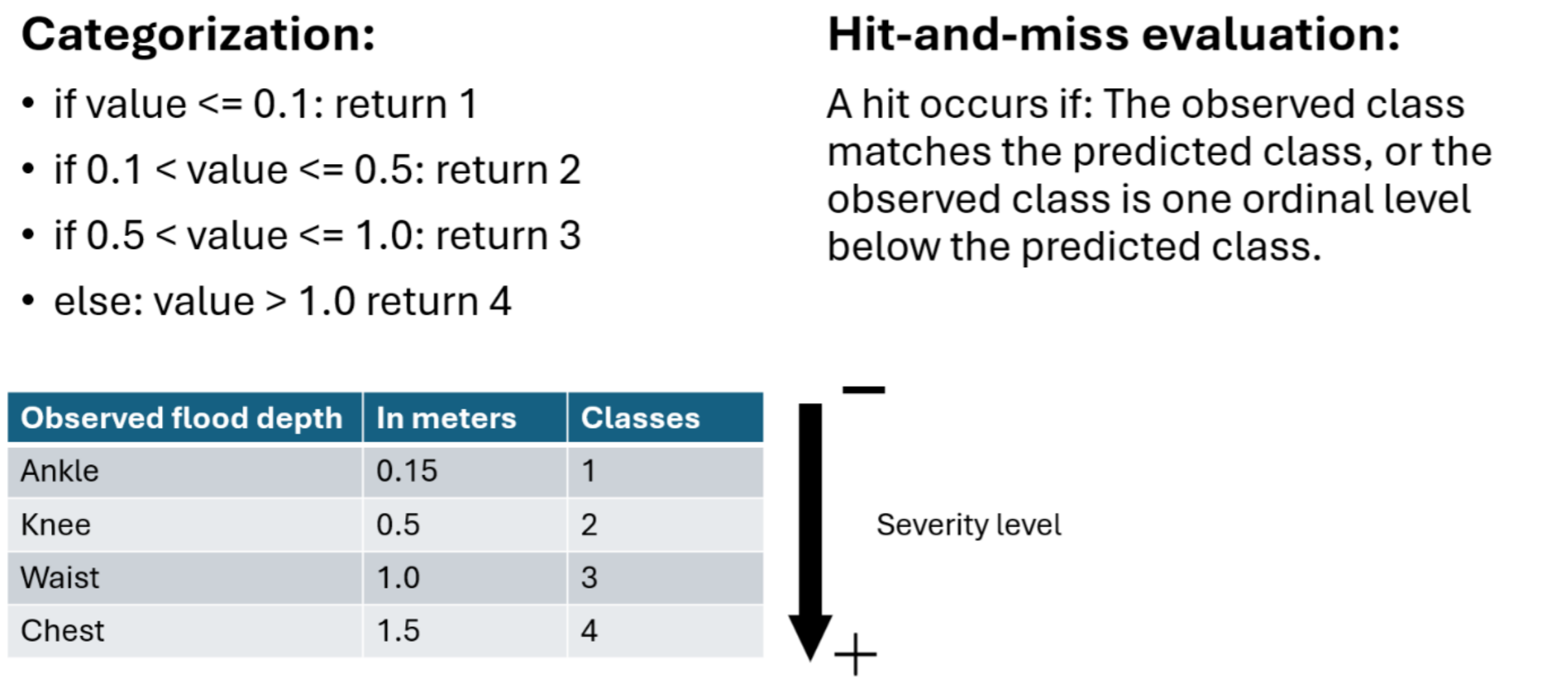

Supplementary Figure 10 – Schematic representation of the hit-and-miss evaluation comparing observed and modelled ordinal flood-depth classes. *The left side shows the categorization of modelled flood depth into four ordinal classes: ankle, knee, waist, and chest. The table defines the representative observed flood-depth values in metres and the corresponding class numbers. The right side illustrates the hit-and-miss rule.*

Content Analysis

This section outlines the systematic approach undertaken to analyze flood observation data. Following the surveys, a total of 338 flood observation points were collected. These raw data points underwent multi-step cleaning process to ensure quality and consistency for analysis. These were to: 1) merge all points to a single table; 2) remove incomplete and ambiguous responses by dropping records without impacts (10 points) and without clear content, either with causing factors or quality of rainfall, (18). Supplementary Table 9 shows common examples that were removed. We minimized the number of removed points as much as possible by resourcing to a secondary analysis based on the workshop discussions and inferred 7 unclear reported impacts in Old Fadama (Accra) and 7 in Mathare (Nairobi); 3) synonyms, capitalization and phrasing issues were fixed to facilitate consistent grouping of impacts; 4) records with two or more impacts (8%) were individually inspected and three overarching themes were identified: Destruction of Structures to Displacement, to Loss of Life or to Geophysical Hazards. Based on insights from local workshops, it was determined that displacement has only been reported as indirect impact of destruction of properties and, similarly, geophysical hazards (erosion and landslide) as direct impacts after floods, thus, they were incorporated to single impacts “Displacement” and “Environmental Issues”, respectively. However, as loss of life could occur independently of or in conjunction to destruction of properties, two distinct categories were maintained “Loss of life” and “Destruction of Property & Loss of Life”. This process resulted in nine standardized flood impact themes, which formed the basis for all subsequent thematic analysis (Supplementary Figure 11). The detailed codebook for each theme is available on 10.5281/zenodo.15648813.

Sample of remarks	Coding	Action
Lack of drainage	Not an impact	Removed
Drainage was done by the community	Not an impact	Removed
Floods were heavier last year	Not an impact	Removed
Accountability	Not an impact	Removed
The flood doesn’t floor	Unclear	Removed

Supplementary Table 9 - Non-impact or unclear survey remarks were removed from the thematic content analysis. *The table provides examples of participant remarks that were excluded during cleaning of the flood-impact dataset. The first column shows sample remarks, the second column reports the coding decision, and the third column reports the action taken.*

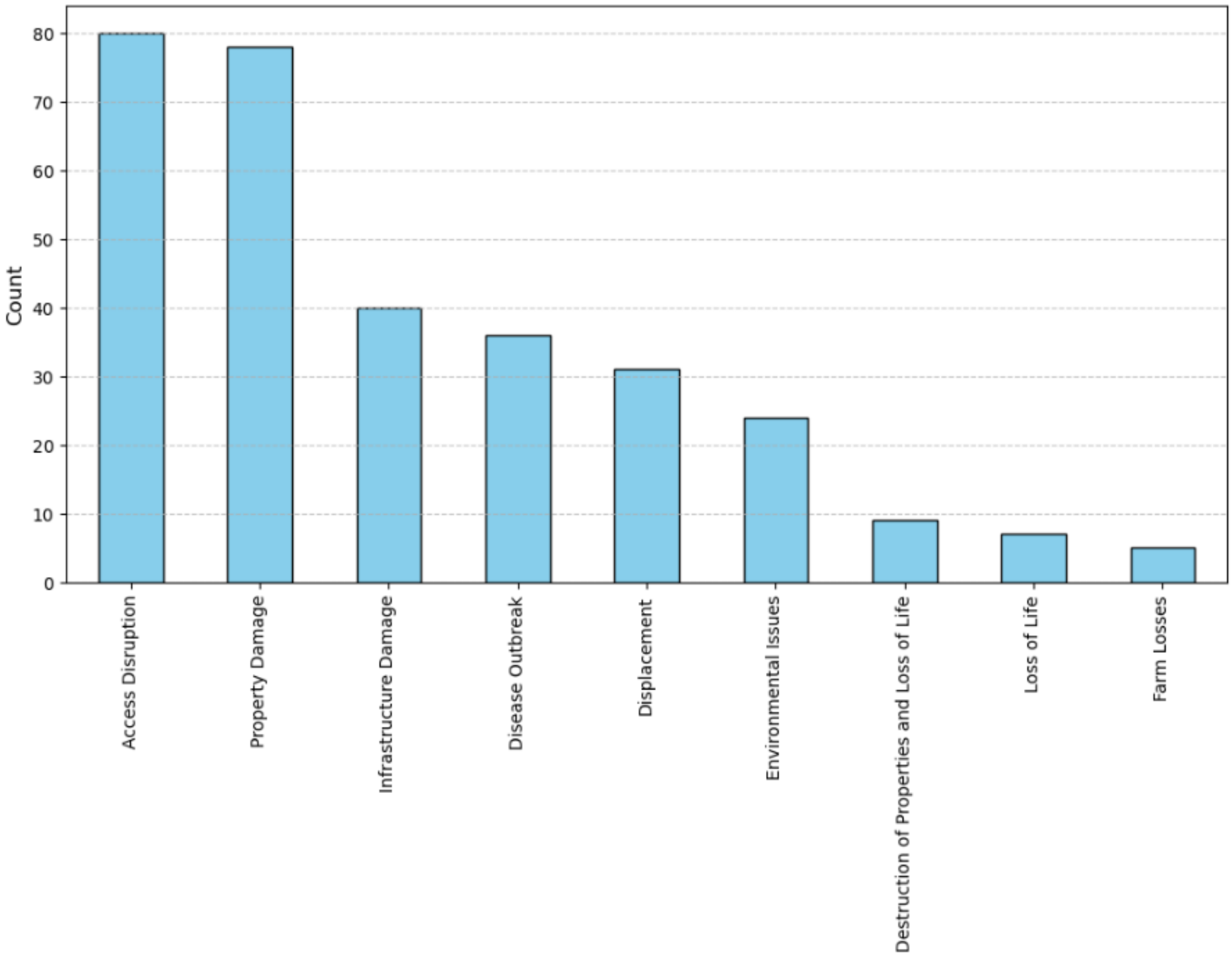

Supplementary Figure 11 - Frequency of standardized themes used in content analysis. *The bar chart shows the frequency of standardized flood-impact themes derived from the cleaned citizen-science observation records. The x-axis lists the final impact themes, and the y-axis shows the number of records assigned to each theme. Light-blue vertical bars represent the count for each theme.*

References

1. MEF. *NATIONAL CLIMATE CHANGE ACTION PLAN (NCCAP) 2018-2022*. (2018).
2. EPA. *Ghana's Fourth National Communication to the United Nations Framework Convention on Climate Change (UNFCCC): National Climate Change Report*. (2020).
3. Elias, P. & de Albuquerque, J. P. Data and the Localization of Sustainable Development Goals in Africa: The Case of SDG 11 in Lagos and Accra. 115–131 (2022). doi:10.1007/978-3-030-95979-1_8
4. Kuffer, M. *et al.* Data Are Power: Addressing the Power Imbalance Around Community Data with the Open-Access Data4HumanRights Curriculum. *Soc. 2025, Vol. 15, Page 29* **15**, 29 (2025).
